# Indian Medicinal Plants and Formulations and Their Potential Against COVID-19–Preclinical and Clinical Research

**DOI:** 10.3389/fphar.2020.578970

**Published:** 2021-03-02

**Authors:** Sayeed Ahmad, Sultan Zahiruddin, Bushra Parveen, Parakh Basist, Abida Parveen, Gaurav, Rabea Parveen, Minhaj Ahmad

**Affiliations:** ^1^ Bioactive Natural Product Laboratory, Department of Pharmacognosy and Phytochemistry, School of Pharmaceutical Education and Research, Jamia Hamdard (Deemed University), New Delhi, India; ^2^ Centre for Translational and Clinical Research, Jamia Hamdard (Deemed University), New Delhi, India; ^3^ Department of Biosciences, Jamia Millia Islamia (Central University), New Delhi, India; ^4^ Department of Surgery, School of Unani Medical Education and Research, Jamia Hamdard (Deemed University), New Delhi, India

**Keywords:** COVID-19, AYUSH medicine, indian medicinal plants, indian traditional medicine, immunomodulators, antiviral agents

## Abstract

The cases of COVID-19 are still increasing day-by-day worldwide, even after a year of its first occurrence in Wuhan city of China. The spreading of SARS-CoV-2 infection is very fast and different from other SARS-CoV infections possibly due to structural differences in S proteins. The patients with severe diseases may die due to acute respiratory distress syndrome (ARDS) caused by systemic inflammatory reactions due to the excessive release of pro-inflammatory cytokines and chemokines by the immune effector cells. In India too, it is spreading very rapidly, although the case fatality rate is below 1.50% (https://www.statista.com), which is markedly less than in other countries, despite the dense population and minimal health infrastructure in rural areas. This may be due to the routine use of many immunomodulator medicinal plants and traditional AYUSH formulations by the Indian people. This communication reviews the AYUSH recommended formulations and their ingredients, routinely used medicinal plants and formulations by Indian population as well as other promising Indian medicinal plants, which can be tested against COVID-19. Special emphasis is placed on Indian medicinal plants reported for antiviral, immunomodulatory and anti-allergic/anti-inflammatory activities and they are categorized for prioritization in research on the basis of earlier reports. The traditional AYUSH medicines currently under clinical trials against COVID-19 are also discussed as well as furtherance of pre-clinical and clinical testing of the potential traditional medicines against COVID-19 and SARS-CoV-2. The results of the clinical studies on AYUSH drugs will guide the policymakers from the AYUSH systems of medicines to maneuver their policies for public health, provide information to the global scientific community and could form a platform for collaborative studies at national and global levels. It is thereby suggested that promising AYUSH formulations and Indian medicinal plants must be investigated on a priority basis to solve the current crisis.

## 1 Introduction

A novel coronavirus-induced pneumonia, which was later called coronavirus disease 2019 (COVID-19), has rapidly increased to an epidemic scale and affected whole human population globally (WHO, 2020a). In India, the first case of COVID-19 was an imported case from Wuhan, China on January 30, 2020 traced in Kerala (Sahasranaman and Kumar, 2020) and the death rate of COVID-19 in India is 1.45%, as of 12th December, 2020 (Worldometers, 2020). Severe acute respiratory syndrome-related coronavirus (SARS-CoV-2) has become a pandemic hazard to global public health worldwide.

Coronaviruses (CoVs) are large viruses comprising of four genera, namely alpha, beta, gamma, and delta. The beta-coronavirus class includes severe acute respiratory syndrome (SARS) virus (SARS-CoV), Middle East respiratory syndrome (MERS) virus (MERS-CoV), and the COVID-19 causative agent SARS-CoV-2. (Li G. et al., 2020). The novel SARS-CoV-2 is a beta CoV that shows 88% similarity to two bat-derived SARS-like CoVs (bat-SL-CoVZC45 and bat-SL-CoVZXC21), about 50% identical to the sequence of MERSCoV, and 70% similarity in genetic sequence to SARS-CoV (Cheng and Shan, 2020). Although there is an extremely high resemblance between SARS-CoV and the novel SARS-CoV-2, the SARS-CoV-2 is spreading rapidly as compared to the SARS-CoV, which may be explained by structural differences in the S proteins (Rabaan et al., 2020).

The SARS-CoV-2 S protein has been found as a significant determinant of virus entry into host cells using angiotensin converting enzyme 2 (ACE2) receptor similar to SARS-CoV. Whereas the binding affinity of virion S glycoprotein and ACE2 is reported to be 10–20 folds higher in SARS-CoV-2 as compared to that of SARS-CoV (Song et al., 2018).

Severe cases of COVID-19 are reported to have increased plasma concentrations of pro-inflammatory cytokines, including interleukins (IL-6 and IL-10), tumor necrosis factor (TNF)-α granulocyte-colony stimulating factor (G-CSF), monocyte chemoattractant protein 1 (MCP1), and macrophage inflammatory protein (MIP)1α (Yuki et al., 2020). Akin to the common viral infections, the antibody profile against the SARS-CoV virus manifests a typical pattern of IgM and IgG antibody production. The IgG antibody is believed to play a protective role, as the SARS-specific IgG antibodies last for a longer time while IgM antibodies disappear at the end of 12 weeks. The latest reports show a significant reduction in the number of CD4^+^ and CD8^+^ T cells in the peripheral blood of SARS-CoV-2-infected patients, besides activation of other pro-inflammatory cytokines such as nuclear factor-κB (NF-κB), interferon regulatory factor 3 (IRF3) and type I Interferons (IFN-α/β) (Li G. et al., 2020). A recent report shows that many patients died from acute respiratory distress syndrome (ARDS) caused by the cytokine storm, which is a deadly uncontrolled systemic inflammatory response resulting from the release of large amounts of pro-inflammatory cytokines and chemokines by immune effector cells in SARS-CoV infection (Guo et al., 2020).

Although the pathogenesis of COVID-19 is still not clear, patients with COVID-19 show non-specific symptoms ranging from no symptoms (asymptomatic) to severe pneumonia and death. However, the most common symptoms include fever, non-productive cough, dyspnea, myalgia, fatigue, diarrhea, lung damage, normal or decreased leukocyte counts, and radiographic evidence of pneumonia, which are similar to the symptoms of SARS-CoV and MERS-CoV infections (WHO, 2020b; Rothan and Byrareddy, 2020). Complications include ARDS, acute heart injury, and secondary infections (Guo et al., 2020).

The present conventional strategy of the disease control includes isolation of cases and tracing their contacts, providing optimal care to these infected cases, reducing chances of secondary infections by early diagnosis, and rapid development of effective diagnostic, preventive and therapeutic strategies, including vaccines (WHO, 2020b). The treatment approach for COVID-19 is supportive care, which is supplemented by the combination of broad-spectrum antibiotics, antivirals, corticosteroids and convalescent plasma (Yang et al., 2020).

Scientists are working hard to develop effective treatments. As of October 18, 2020, more than 3611 clinical trials (with more than 100 complementary medicines) on COVID-19 are either ongoing or enrolling patients, and new ones are being added every day, as the case count skyrockets globally. The drugs being tested range from repurposed flu treatments to failed ebola drugs, to malaria treatments that were first developed decades ago (Lythgoe and Middleton, 2020). There is scale-up development of vaccines across the world by many pharmaceutical companies as well as research organizations. These treatments undergoing trials may require months or years to develop and hit the market, meaning that an immediate treatment or control mechanism should be found, if possible (Table 1).

**TABLE 1:** Details of clinical trials completed on AYUSH drugs for COVID-19 (Source: www.ctri.nic.in).

Ctri No./Treatment details	Study title	Type of trial (design of study) Recruitment status	Remarks
CTRI/2020/04/024883 ZINGIVIR-H	Clinical research on safety and efficacy of ZingiVir-H as an add on therapy in COVID-19 patients	Interventional (Other) Completed	Zingivir H consumption with standard of care in COVID 19 confirmed patients showed a remarkable recovery compared to that of placebo
CTRI/2020/05/025161/Herbal formulation-aayudh advance	To study the effectiveness of herbal formulation - aayudh advance as a supplementary treatment for the corona virus 2019 (COVID-19) infected patients	Interventional (randomized, parallel group, active controlled Trial) Completed	“Aayudh advance”, when given concomitantly with standard of care, was found to be 100% safe, devoid of any drug-drug interaction, effective as virucidal to reduce viral load, and increased the recovery rate when compared to standard of care alone when tested in mild symptomatic COVID-19 patients
CTRI/2020/05/025215/Kabasura kudineer	Effectiveness of siddha medicine, kabasura kudineer and vitamin c-zinc supplementation in the management of mild COVID-19 patients	Interventional (randomized, parallel group Trial) Completed	The role of vitamin C with zinc supplementation in the management of COVID 19 is still not clear. Therefore, study will compare the effect of kabasura kudineer and vitamin C with zinc supplementation in terms of negative conversion of SARS CoV- 2 infection
CTRI/2020/05/025275/Ayurveda rasayana along with conventional guidelines for health care workers	Role of chyawanprash in the prevention of COVID-19 in health care workers	Interventional (randomized, parallel group Trial) Completed	No adverse effect was found in the study
CTRI/2020/05/025276/Ayurveda protocol	Effect of ayurvedic intervention in COVID-19 positive cases	Interventional (single arm Trial) Completed	Ayurveda treatment protocol includes sanshamani, nagaradi kwath, amalaki churna and golden milk improved the strength of the patient
CTRI/2020/05/025397/Purified aqueous extract of cocculus hirsutus (AQCH)	A study to evaluate the effect and safety of a phytopharmaceutical drug in treatment of coronavirus infection	Interventional (randomized, parallel group Trial) Completed	Clinical improvement was observed in covid patients in terms of disease severity
CTRI/2020/05/025425/Chayapanprash (an ayurvedic herbal preparation)	Ayurvedic intervention (chyawanprash) in the prevention of COVID-19 pandemic among health care personnel	Interventional (single arm Trial) Completed	This remedy was found to be a possible safe prophylactic remedy for COVID-19
CTRI/2020/06/025527/Amrta karuna syrup	Clinical trial on immunity and antiviral for quarantine patients of COVID-19	Interventional (randomized, parallel group, active controlled Trial) Closed to recruitment	The formulation was found to be immunomodulatory
CTRI/2020/06/025556/Virulina^®^ along with standard treatment protocol	A clinical trial to know the effect of Virulina^®^ along with standard treatment in COVID-19 positive patients	Interventional (randomized, parallel group, placebo controlled Trial) Completed	The formulation was found to boost the immunity of the patients and help ease the symptoms
CTRI/2020/06/025590/Astha 15 capsule	A clinical trial to evaluate safety and efficacy of polyherbal capsule Astha-15 used as an add on therapy with standard care of therapy as an immunity booster in the suspected and COVID-19 diagnosed patients	Interventional (randomized, parallel group, placebo controlled Trial) Completed	A better recovery rate was observed
CTRI/2020/06/025592/Immunity kit	Use of herbal medicine like tulsi, amruth (giloy), turmeric, ashwagandha as add on treatment in COVID-19 patients	Interventional (single arm Trial) Completed	Upon using the ayurvedic formulation as add on treatment, the recovery was better in terms of signs and symptoms of COVID-19 patients
CTRI/2020/06/026221/Arogya Kashayam-20	Intervention of ayurvedic medicine (arogya kashayam) in COVID-19 positive cases (asymptomatic and mild symptomatic)	Interventional (randomized, parallel group, active controlled Trial) Completed	The unani regimen was found to be effective against the mild symptoms of covid 19
CTRI/2020/06/026227/Khameera marwareed Tiryaq-e-Arba Unani joshanda/decoction behidana (*Cydonia oblonga*) 3 gm, unnab (*Zizyphus jujube*) 5 in number, sapistan (*Cordia myxa*) 9 in numbers	A study on unani regimen for prevention of high/moderate risk population of COVID-19	Interventional (non-randomized, multiple arm Trial) Completed	Improvement was found in immune status of covid patients
CTRI/2020/06/025801/Tab. Bresol and tab. Septilin	Role of herbal immunomodulators in mild COVID-19 confirmed cases	Interventional (randomized, parallel group, active controlled Trial) Completed	Use of herbal immunomodulators as add on treatment, improved the recovery rate of COVID-19 patients
CTRI/2020/07/026337/Add-on personalized ayurveda intervention to ICMR guideline on Covid-19	The COVID-19 study with ayurveda add-on to ICMR guideline	Interventional (randomized, parallel group trial) completed	Efficacy of treatment was measured in terms of average stay of patients in the hospital to become covid negative
CTRI/2020/07/026371/1. Kabasura kudineer 2.Shakti drops 3.Turmeric plus tablets	Kabasura kudineer, shakti drops and turmeric plus in the management of COVID-19	Interventional (Others) Completed	Better recovery rate was observed in terms of signs and symptoms of stage 1 and 2 of COVID-19 cases on addition of ayurvedic medicines, thereby improving the quality life of stage 1 and 2 of COVID-19 patients
CTRI/2020/07/026433/1. Dashamula kwatha and pathyadi kwatha with trikatu churna 2. Sansamani vati 3. AYUSH 64 4. Yastimadhu Ghanavati	Effect of ayurveda medicine in COVID-19 mild symptoms	Interventional (randomized, parallel group, active controlled Trial) Completed	No adverse reaction was observed and improvement in signs and symptoms
CTRI/2020/07/026570/Cap. IP	Safety and efficacy of ayurvedic capsule in mild to moderate COVID-19 infection	Interventional (randomized, parallel group Trial) Completed	Improvement was observed in respiratory symptoms of covid patients

Considering the current situation, various treatment modalities have been well-thought-out, including traditional medicine, which has been widely used during the past epidemic outbreaks, including SARS and H1N1 influenza (Luo et al., 2020). Until now three countries including India, China, and South Korea, have issued guidelines on traditional regimens for the prevention and management of COVID-19 (Ang et al., 2020).

The Indian Traditional System of Medicine is one of the oldest systems of medical practice in the world and has played an essential role in providing health care service to human civilization, right from its inception. India has the exclusive distinction of its own recognized traditional medicine; Ayurveda, Yoga, Unani, Siddha, and Homoeopathy (AYUSH) (Adhikari and Paul, 2018). These systems are based on definite medical philosophies and represent a way of achieving a healthy lifestyle with conventional and established ideas on the prevention of diseases and the promotion of health. The basic treatment approach of all these systems is holistic and the pharmacological modalities are based on natural products of plants, animals, or mineral origin. Given this, there is a resurgence of interest in AYUSH systems, which have helped the nation in the pandemic crisis due to plague, cholera, Spanish flu, etc. in the past. Hence, by repurposing the traditional uses of Indian medicinal plants and formulations, new treatment options can be identified to combat the current deadly pandemic. In view of the COVID-19 outbreak, the entire human race across the globe is perturbed. While there is no medicine for COVID-19 as of now, it is imperative to take preventive measures such as practicing self-hygiene, social distancing and boosting immunity. Many safe traditional formulations of AYUSH, which are well known immunity modulators, have been used for centuries in respiratory disorders and in allergic conditions. The Ministry of AYUSH (Govt of India) has listed out such formulations and recommended their use as a prophylactic measure in red zones, containment zones, as well as for corona warriors. Many of them are now under clinical trial in COVID-19 patients (Table 1).

**TABLE 2:** AYUSH recommended prophylactic approach through Ayurvedic formulations. Ref: AYUSH Ministry of Health Corona Advisory-D.O. No. S. 16030/18/2019- NAM; dated: 06th March, 2020. Ref: AYUSH Ministry of Health Corona Advisory -F.No. Z 25.23/09/2018–2020-DCC (AYUSH); dated: 24th April, 2020.

Name of the formulation	Composition	Proof of activity related to COVID-19	References
**Anuthaila**	*Leptadenia* reticulate (Retz.) Wight and Arn. (root/stem bark)	A,C	Pravansha et al. (2012), Mohanty et al. (2015)
	*Cedrus deodara* (Roxb. ex D.Don) G.Don (stem)	B	Raghavendhar et al. (2019)
	*Vetiveria zizanioides* (L.) Nash (root)	B	Lavanya et al. (2016)
	*Ocimum sanctum* L. (leaves)	A,B,C	Goel et al. (2010), Ghoke et al. (2018), Soni et al. (2015)
	*Berberis aristata* DC. (bark)	A,B,C	Yan et al. (2018), Wang et al. (2017), Kumar et al. (2016)
	*Glycyrrhiza glabra* L. (root rhizome)	A,B,C	Mitra Mazumder et al. (2012), Ashraf et al. (2017), Patel et al. (2009)
	*Cyperus rotundus* L. (rhizome)	A,B,C	Soumaya et al. (2013), Xu et al. (2015), Jin et al. (2011)
	*Asparagus racemosus* Willd. (root)	A	Gautam et al. (2009)
	*Aegle marmelos* (L.) Correa (stem bark)	A,C	Patel and Asdaq (2010), Kumari et al. (2014)
	*Solanum indicum* L. (leaves)	C	Kaunda and Zhang (2019)
	*Solanum xanthocarpum* Schrad. and Wendl (fruit)	B	Kumar and Pandey (2014)
	*Uraria picta* (jacq.) DC. (whole plant)	C	Nagarkar et al. (2013)
	*Embelia ribes* Burm.f. (fruit)	B,C	Mahendran et al. (2011)
	*Cinnamomum verum* J.Presl. (bark)	A,B,C	Niphade et al. (2009), Brochot et al. (2017), Kandhare et al. (2013)
	*Elettaria cardamomum* (L.) Maton (fruit)	B	Rahman et al. (2017)
	*Vitex negundo* L. (leaves)	A,B,C	Lad et al. (2016), Kannan et al. (2012), Chattopadhyay et al. (2012)
	*Sesamum indicum* L. (seed oil)	A,C	Khorrami et al. (2018), Nagpurkar and Patil (2017)
**Agasthaya hareetaki**	*Aegle marmelos* (L.) Correa (root/stem bark)	A,C	Patel and Asdaq (2010), Kumari et al. (2014)
	*Oroxylum indicum* (L.) Kurz (root/stem bark)	B	Zaveri et al. (2008)
	*Gmelina arborea* Roxb. (root/stem bark)	B	Panda et al. (2017)
	*Stereospermum suaveolens* (Roxb.) DC. (root/stem bark)	C	Balasubramanian et al. (2010)
	*Premna mucronta* Roxb. (root/stem bark)	A,C	Dianita and Jantan (2017)
	*Desmodium gangeticum* (L.) DC. (whole plant)	A	Gulati et al. (2002)
	*Uraria picta* (jacq.) DC. (whole plant)	C	Nagarkar et al. (2013)
	*Solanum indicum* L. (whole plant)	C	Kaunda and Zhang (2019)
	*Solanum surattense* Burm.f. (whole plant)	C	Kaunda and Zhang (2019)
	*Tribulus terrestis* L. (whole plant)	B,C	Malik et al. (2018), Kang et al. (2017)
	*Mucuna pruriens* (L.) DC. (seed)	B,C	Lampariello et al. (2012)
	*Convolvulus pluricaulis* Choisy (whole plant)	A,B,C	Agarwal et al. (2014)
	*Hedychium spicatum* Sm. (rhizome)	A,C	Uttara and Mishra (2009), Ghildiyal et al. (2012)
	*Sida cordifolia* L. (root)	A,C	Tekade et al. (2008), Singh S. et al. (2011)
	*Piper chaba* Hunter (fruit)	C	Sireeratawong et al. (2012)
	*Achyranthes aspera* L. (root)	A,B,C	Narayan and Kumar (2014), Mukherjee et al. (2013), Khuda et al. (2013)
	*Piper longum* L. (root)	A,B,C	Tripathi et al. (1999), Jiang et al. (2013), Kaushik et al. (2012)
	*Plumbago zeylanica* L. (root)	B	Gebre-Mariam et al. (2006)
	*Clerodendron serratum* Spr. (root)	A	Juvekar et al. (2006)
	*Inula racemosa* Hook.f. (root)	A,C	Mishra et al. (2016), Vadnere et al. (2009)
	*Hordeum vulgare* L. (seed)	C	Gul et al. (2014)
	*Terminalia chebula* Retz. (pulp)	A,B,C	Shivaprasad et al. (2006), Kesharwani et al. (2017), Haq et al. (2013)
**Samshamani vati**	*Tinospora cardifolia* (Willd.) Miers (stem)	A,B,C	Alsuhaibani and Khan (2017), Pruthvish and Gopinatha (2018), Tiwari et al. (2014)
**AYUSH-64**	*Alstonia scholaris* (L.) R.Br. (bark)	A,B,C	Iwo et al. (2000), Antony et al. (2014), Zhao et al. (2017)
	*Picrorhiza kurroa* Royle ex Benth. (rhizome)	A,B,C	Sharma et al. (1994), Win et al. (2019), Sehgal et al. (2013)
	*Swertia chirayita* (Roxb.) H.Karst. (whole plant)	B,C	Woo et al. (2019), Khan et al. (2012)
	*Caesalpinia crista* L. (seed pulp)	C	Ramesh et al. (2014)
**AYUSH kwath**	*Ocimum sanctum* L. (leaves)	A,B,C	Goel et al. (2010), Ghoke et al. (2018), Soni et al. (2015)
	*Cinnamomum verum* J.Presl. (stem bark)	A,B,C	Niphade et al. (2009), Brochot et al. (2017), Kandhare et al. (2013)
	*Zingiber officinale* Roscoe (rhizome)	A,B,C	Zhou et al. (2006), Chang et al. (2013), Khan et al. (2015)
	*Piper nigrum* L. (fruit)	A,B,C	Majdalawieh and Carr (2010), Mair et al. (2016), Tasleem et al. (2014)

Note: A = Immunomodulators; B = Antiviral; C = Anti-allergic/Anti-asthmatic/Anti-inflammatory/Respiratory disorders.

**TABLE 3:** AYUSH recommended prophylactic approach through Unani formulation. Ref: AYUSH Ministry of Health Corona Advisory–D.O. No. S. 16030/18/2019- NAM; dated: 06th March, 2020.

Name of the formulation	Composition	Proof of activity related to COVID-19	References
Arq-e-Ajeeb	Camphor	B,C	Chen et al. (2013), Ziment and Tashkin (2000)
	Menthol	B,C	Taylor et al. (2020), Ziment and Tashkin (2000)
	Thymol	C	Al-Khalaf (2013)
Asgandh safoof	*Withania somnifera* (L.) Dunal (root)	A,B,C	Rasool and Varalakshmi (2006), Pant et al. (2012), Sahni and Srivastava (1993)
Habb-e-Bukhar	*Cinchona officinale* L. (bark)	B	Devaux et al. (2020)
	*Tinospora cordifolia* (Willd.) Miers (stem)	A,B,C	Alsuhaibani and Khan (2017), Pruthvish and Gopinatha (2018), Tiwari et al. (2014)
	*Bambusa bambos* (L.) Voss (stem)	A	Sriraman et al. (2015)
	*Acacia arabica* (Lam.) Willd. (gum)	C	Roqaiya et al. (2015)
Habb-e-Hindi zeeqi	*Aconitum chasmanthum* Stapf ex Holmes (root)	C	Alamgeer et al. (2018)
	*Calotropis procera* (Aiton) W.T.Aiton (root)	A,C	Bagherwal (2011), Arya and Kumar (2005)
	*Zingiber officinale* Roscoe (rhizome)	A,B,C	Zhou et al. (2006), Chang et al. (2013), Khan et al. (2015)
Habb-e-Mubarak	*Myrica esculenta* Buch.-Ham. ex D.Don (stem bark)	A,C	Kabra et al. (2019)
	*Caesalpinia bonduc* (L.) Roxb. (cotyledon)	A,C	Shukla et al. (2010), Arunadevi et al. (2015)
Khamira-e-Banafsa	*Viola odorata* L. (flower)	B,C	Gerlach et al. (2019), Koochek et al. (2003)
Khameera marwareed	*Mytilus margaritiferus* (pearl)	A	Khan et al. (2009), Beaulieu et al. (2013)
	*Bambusa bambos* (L.) Voss (stem)	C	Muniappan and Sundararaj (2003)
	*Vateria indica* L. (gum)	B,C	Meena and Ramaswamy (2015)
	*Santalum album* L. (stem)	B,C	Paulpandi et al. (2012), Gupta and Chaphalkar (2016)
	*Rosa × damascena* Mill. (flower)	B,C	Mahmood et al. (1996), Boskabady et al. (2011)
Laooq-e-Katan	*Linum usitatissimum* L. (seed)	A,C	Liang et al. (2019), Rafieian-kopaei et al. (2017)
Laooq-e-Sapistan	*Cordia myxa* L. (fruit)	A,B,C	Ali et al. (2015), Rashed (2014), Ranjbar et al. (2013)
	*Ziziphus jujuba* Mill. (fruit)	A,B,C	Yu et al. (2016), Hong et al. (2015), Mesaik et al. (2018)
	*Viola odorata* L. (flower)	B,C	Gerlach et al. (2019), Koochek et al. (2003)
	*Althea officinalis* L. (seed)	C	Bonaterra et al. (2020)
	*Cassia fistula* L. (seed)	A,B,C	Laxmi (2015), Indrasetiawan et al. (2019), Antonisamy et al. (2019)
	*Cassia angustifolia* M. Vahl (leaves)	A	Jassim and Naji (2003)
	*Fraxinus ornus* L. (flower)	C	Al-Snafi (2018)
	*Prunus amygdalus* Batsch (seed oil)	B,C	Musarra-Pizzo et al. (2019), Masihuddin et al. (2019)
Roghan-e-Baboona	*Matricaria chamomilla* L. (flower)	A,C	Amirghofran et al. (2000), Singh O. et al. (2011)
Sarbat-e sadr	Bombyx mori (cocoons)	A	Soumya et al. (2019)
	*Ziziphus jujuba* Mill. (fruit)	A,B,C	Yu et al. (2016), Hong et al. (2015), Mesaik et al. (2018)
	*Tachyspermum ammi (L.) Sprague* (seed)	A,B	Shruthi et al. (2017), Roy et al. (2015)
	*Glycyrrhiza glabra* L. (root)	A,B,C	Mitra Mazumder et al. (2012), Ashraf et al. (2017), Patel et al. (2009)
	*Foeniculum vulgare* Mill. (fruit)	C	Rather et al. (2016)
	*Adhatoda vasica* Nees (leaves)	A,B,C	Vinothapooshan and Sundar (2011), Singh et al. (2010), Gibbs (2009)
	*Onosma bracteatum* Wall. (leaves)	C	Patel et al. (2011)
	*Malva sylvestris* L. (seed)	C	Martins et al. (2017)
	*Hyssopus offloinalis* L. (whole plant)	B	Behbahani (2009)
	*Ficus carica* L. (fruit)	A,B,C	Patil et al. (2010), Camero et al. (2014), Abe (2020)
	*Cordia myxa* L. (fruit)	C	Oza and Kulkarni (2017)
	*Papaver somniferum* L. (flower)	B,C	Chattopadhyay and Naik (2007)
	*Onosma bracteatum* Wall. (flower)	C	Patel et al. (2011)
Sharbat-e-Toot siyah	*Morus nigra* L. (fruit)	A,C	Lim and Choi (2019)
Triyaq-e-Araba	*Laurus nobilis* L. (berries)	A	Aurori et al. (2016)
	*Bergenia ciliata* (haw.) Sternb. (stem)	A	Rajbhandari et al. (2009)
	*Aristolochia indiaca* L. (root)	C	Mathew et al. (2011)
	*Commiphora myrrha* (Nees) Engl. (gum)	C	Su et al. (2015)

Note: AProvide the references Mallik and nayak (2014), Sengottuvelu et al. (2012), and Weili et al. (2011)= Immunomodulators; B = Antiviral; C = Anti-allergic/Antiasthmatic/Anti-inflammatory/Respiratory disorders.

**TABLE 4:** AYUSH recommended prophylactic approach through formulations of Siddha system of medicine. Ref: AYUSH Ministry of Health Corona Advisory–D.O. No. S. 16030/18/2019-NAM; dated: 06th March, 2020.

Name of the formulation	Composition	Proof of activities related to COVID-19	References
Ahatodai manapagu (siddha)	*Adhatoda vasica* Nees (leaves)	A,B,C	Vinothapooshan and Sundar (2011), Singh et al. (2010), Gibbs (2009)
	*Saccharum officinarum* L.	C	Cheavegatti-Gianotto et al. (2011)
Kabasura kudineer (siddha)	*Zingiber officinale Roscoe* (rhizome)	A,B,C	Zhou et al. (2006), Chang et al. (2013), Khan et al. (2015)
	*Piper longum* L. (fruit)	A,B,C	Tripathi et al. (1999), Jiang et al. (2013), Kaushik et al. (2012)
	*Syzygium aromaticum* (L.) Merr. and L.M. Perry (fruit)	A,C	Dibazar et al. (2015), Chniguir et al. (2019)
	*Tragia involucrate* L. (leaves)	B,C	Kumar et al. (2019), Alagar Yadav et al. (2015)
	*Anacyclus pyrethrum* (L.) Lag. (root)	A,B	Sharma et al. (2010), Kumar et al. (2019)
	*Adhatoda vasica Nees (leaves)*	A,B,C	Vinothapooshan and Sundar (2011), Singh et al. (2010), Gibbs (2009)
	*Tinospora cordifolia* (Willd.) Miers (stem)	A,B,C	Alsuhaibani and Khan (2017), Pruthvish and Gopinatha (2018), Tiwari et al. (2014)
	*Andrographis paniculata* (Burm.f.) Nees (whole plant)	A,B,C	Wang et al. (2010), Wintachai et al. (2015), Bao et al. (2009)
	*Sida acuta* Burm.f. (root)	C	Arciniegas et al. (2017)
	*Cyperus rotundus* L. (rhizome)	A,B,C	Soumaya et al. (2013), Xu et al. (2015), Jin et al. (2011)
	*Terminalia chebula* Retz. (pulp)	A,B,C	Shivaprasad et al. (2006), Kesharwani et al. (2017), Haq et al. (2013)
Nilavembu kudineer (siddha)	*Andrographis paniculata* (Burm.f.) Nees (whole plant)	A,B,C	Wang et al. (2010), Wintachai et al. (2015), Bao et al. (2009)
	*Plectranthus vettiveroides* (Jacob) N.P.Singh and B.D.Sharma (root)	A,B	Kavinilavan et al. (2017)
	*Vetiveria zizanioides* (L.) Nash (root)	B	Lavanya et al. (2016)
	*Zingiber officinale* Roscoe (rhizome)	A,B,C	Zhou et al. (2006), Chang et al. (2013), Khan et al. (2015)
	*Piper Nigrum* L. (fruit)	A,B,C	Majdalawieh and Carr (2010), Mair et al. (2016), Tasleem et al. (2014)
	*Cyperus rotundus* L. (rhizome)	A,B,C	Soumaya et al. (2013), Xu et al. (2015), Jin et al. (2011)
	*Santalum album* L. (stem)	B,C	Paulpandi et al. (2012), Gupta and Chaphalkar (2016)
	*Trichosanthes cucumerina* L*.* (whole plant)	B,C	Kumar et al. (2019), Arawwawala et al. (2010)
	*Mollugo cerviana* (L.) Ser. (whole plant)	A,B,C	Ferreira et al. (2003), Jain et al. (2019), Sadique et al. (1987)

Note: A = Immunomodulators; B = Antiviral; C = Anti-allergic/Antiasthmatic/Anti-inflammatory/Respiratory disorders.

Similarly, there are many medicinal plants indigenous to India and used in the Indian Systems of Medicine which have been reported as potent antiviral with immunomodulatory and anti-allergic/anti asthmatic activities. Many of these medicinal plants are also an integral part of several traditional formulations that have been in use for a long time.

This review discusses the possible alternative strategies for the management of the SARS-CoV-2 infection by reducing its morbidity in patients as an adjuvant to modern therapy and also by providing prophylactic management. Further, potential testing targets of botanicals from Indian medicinal plants need to be explored against SARS-CoV-2 infection and categorized on a priority basis in view of their reported antiviral, immunomodulatory and other related activities.

## 2 Potential Traditional Indian/AYUSH Formulations for the Management of COVID-19

There is plenty of data supporting the effectiveness of herbs in treating the viral infection. For instance, in controlling the contagious disease spread in the Guangdong Province of China during the 2003 SARS outbreak (Zhang et al., 2020). There are convincing pieces of evidence to establish that traditional Chinese medicine (TCM) has favorable effect in the treatment or prevention of SARS (Yang et al., 2020). A combination of modern and traditional therapy might reduce the severity of the disease, intensity of symptoms, death rate, and side effects. Similar are the observations for *Shuanghuanglian* (A Chinese medicine) a liquid composed of a blend of honeysuckle, Chinese skullcap, and forsythia, which is claimed to have antiviral, antibacterial, and immunomodulatory effects (https://www.bioworld.com/). Since AYUSH encompasses five different systems of medicine, rich in a variety of traditional formulations, it is likely to have a better chance than other systems to come up with a satisfactory solution to the COVID-19 crisis.

Ayurveda means ‘Science of life’. It provides a complete system to have a long and healthy life. It is derived from the concepts of “*Dinacharya”* - daily regimes and “*Ritucharya*” - seasonal regimes to maintain a healthy life. Uplifting and maintaining the immunity is duly emphasized across the Ayurveda’s classical scriptures.

The Unani system of medicine, known as Greco-Arab Medicine, is built on the four conditions of living (hot, sodden, frosty, and dry) and four humors of Hippocratic hypothesis namely, blood, yellow bile, dark bile, and mucus. Epidemics, referred to as waba in the Unani system of medicine, are thought to occur if any contagion or ajsam-i -khabitha, finds a place in air and water. Furthering the view, Ibn-e-Sina (980–1035 CE) stated that epidemics spread from one person to another, and one city to another ‘like a message’ (Sina, 1878).

AYUSH systems of medicine propagate general preventive measures aimed at preventing the spread of infection such as social distancing, hygiene and anti-septic measures (sanitization of surroundings), improvement of immunity, and promotion of general health (dietary modifications and herbal drugs). The present article elucidates some traditional Indian AYUSH formulations with proven antiviral, anti-asthmatic, and immunomodulatory activities, however their role in combating COVID-19 needs to be established. Clinical trials of AYUSH medicines like Ashwagandha, Yashtimadhu, Guduchi, Pippali, and AYUSH-64 on patients, health workers, and those working in high-risk areas have been initiated in India by the Ministry of AYUSH, Ministry of Health and Family Welfares, and the Council of Scientific and Industrial Research (CSIR) with the technical support of Indian Council of Medical Research (ICMR) (Table 1).

## 3 AYUSH Recommendations for Management of COVID-19

Based on the different systems of Indian Medicine, separate recommendations have been issued from time to time from the Ministry of AYUSH (Government of India) for the management of COVID-19. These different approaches are being followed by the Hospitals as per their specialization, mainly as adjuvants to modern medicine, which could be potentially relevant for COVID 19 treatment. Details of recommended formulations are described below and depicted in Table 2 (Ayurveda), Table 3 (Unani) and Table 4 (Siddha).

### 3.1 Ayurvedic Approaches

#### 3.1.1 AYUSH Kwath

Ministry of AYUSH promotes the use of AYUSH kwath, which is a ready-made formulation for health promotion of the masses. The formulation is made of four herbs *Ocimum sanctum* L. leaves, *Cinnamomum verum* J. Presl. stem barks, *Zingiber officinale* Roscoe rhizomes and *Piper nigrum* L. fruits. The formulation is sold in the market with different names like ‘AYUSH Kwath’, ‘AYUSH Kudineer’ or ‘AYUSH Joshanda’. It is available in powder and tablet forms in the market. These herbs are reported to boost immunity (Carrasco et al., 2009; Niphade et al., 2009; Alsuhaibani and Khan, 2017; Bhalla et al., 2017) and are active remedies to various viral diseases (Mair et al., 2016; Ghoke et al., 2018; Pruthvish and Gopinatha, 2018).

#### 3.1.2 Samshamani Vati

Samshamani vati (Guduchi ghana vati) is an ayurvedic formulation used in all types of fevers. It is also used as an antipyretic and anti-inflammatory remedy (Patgiri et al., 2014). Samshamani vati is made of aqueous extract of *Tinospora cordifolia* (Willd.) Miers (family Menispermaceae), and reported to be an immunomodulator (More and Pai, 2011) due to the synergistic effect of the various compounds present. It is also effective in various viral diseases (Sachan et al., 2019).

#### 3.1.3 AYUSH-64

AYUSH-64 tablet is composed of *Alstonia scholaris* (L.) R. Br. bark*, Picrorhiza kurroa* Royle ex Benth. rhizomes, *Swertia chirayita* (Roxb.) H. Karst. whole plant, and *Caesalpinia crista* L. seed pulp. Because of its antimalarial activity, AYUSH-64 is considered to be effective among the high-risk coronavirus population. Researchers have reported that each of its constituents is effectively antiviral, anti-asthmatic, and immunoboosting (Sharma et al., 1994; Siddiqui et al., 2012; Sehgal et al., 2013; Panda et al., 2017; Win et al., 2019; Woo et al., 2019).

#### 3.1.4 Agasthaya Hareetaki

Agastya Haritaki Rasayana is a popular ‘Avaleha kalpana’, used in the management of various respiratory infection and comprises more than 15 herbal ingredients. Most of its ingredients showed antiviral, anti-asthmatic, anti-inflammatory, and immunomodulatory activities (Mouhajir et al., 2001; Tripathi and Upadhyay, 2001; Balasubramanian et al., 2007; Vadnere et al., 2009; Patel and Asdaq, 2010; Pathak et al., 2010; Jain et al., 2011; Kumar et al., 2011; Lampariello et al., 2012; Jiang et al., 2013). The above literature suggests the symptomatic management of COVID-19 by Agastya Haritaki.

#### 3.1.5 Anuthaila

Anuthaila consists of about twenty ingredients and out of them *Leptadenia reticulate* (Retz.) Wight and Arn. has been reported in allergic response, treatment of asthma, bronchitis, and throat trouble (Mohanty et al., 2017). Similarly, *Ocimum sanctum* L. is recommended for a wide range of conditions including, cough, asthma, fever, and malaria (Cohen, 2014) and *Sesamum indicum* L. oil for dry cough, asthma, migraine, and respiratory infections (Nagpurkar and Patil, 2017). There are reports on *S. indicum* seeds with *Tachyspermum ammi* (L.) Sprague seeds for dry cough, asthma, lung diseases, and common cold (Patil et al., 2008). On the basis of above literature, Anuthaila justifies its use in corona virus pandemic condition (Table 2).

### 3.2 Unani Approaches

#### 3.2.1 Triyaq-e-Araba

Triyaq-e-Araba is an important Unani formulation used as a detoxifying agent. It contains *Laurus nobilis* L. berries, *Bergenia ciliate* (Haw.) Sternb. stem, *Aristolochia indica* L. roots and *Commiphora myrrha* (Nees) Engl*.* It has been reported by several authors as a potent antiviral agent (Aurori et al., 2016), including against SARS-CoV (Loizzo et al., 2008). Further, *B. ciliata* is found to be effective against the influenza virus-A and herpes simplex virus-1 (HSV-1) (Rajbhandari et al., 2003), whereas its active principal, bergenin, has been found to be effective against hepatitis C virus (HCV) and HIV virus (Ahmad et al., 2018). On the basis of this literature, Triyaq-e-Araba could be one of the effective antiviral medicine and certifies its use against COVID-19.

#### 3.2.2 Roghan-e-Baboona

Roghan-e-Baboona is an Unani remedy utilized as an anti-asthmatic and for the treatment of inflammatory complaints. Flowers of *Matricaria chamomilla* L. are the main ingredient of Roghan-e-Baboona. It is composed of the flowers of *M. chamomilla*, which is found effective for acute viral nasopharyngitis (Srivastava et al., 2010), as well as for sore throat (Kyokong et al., 2002).

#### 3.2.3 Arq-e-Ajeeb

Arq-e-Ajeeb is a liquid preparation that contains thymol, menthol, and camphor. Thymol is a promising candidate for topical application as an antiviral agent for herpetic infections (Lai et al., 2012; Sharifi-Rad et al., 2017). Menthol has been reported as an anti-inflammatory agent (Zaia et al., 2016). The Unani physicians have a very successful history of treating Nazla wabai (Swine flu) using Arq -e-Ajeeb. These studies support the use of Arq-e-Ajeeb for COVID-19.

#### 3.2.4 Khamira-e-Banafsha

Khamira-e-Banafsha is a semi-solid Unani formulation prepared by adding decoction of flowers of *Viola odorata* L. to a base of sugar or sugar with honey and used for cold-cough as expectorant and for the treatment of ailments of respiratory system and chest diseases, bronchitis, whooping cough, fever, expectorant, antipyretic etc. Further, *V. odorata* has been reported to suppress the viral load and increase antiretroviral drug efficacy (Gerlach et al., 2019), decrease the thickness of the alveolar wall, hemorrhage area, and alter the epithelial lining of bronchioles of the lungs (Koochek et al., 2003). The above literature supports its use for the management of COVID-19.

#### 3.2.5 Laooq-e-Sapistan

Laooq-e-Sapistan is a semisolid sugar-based polyherbal Unani formulation extensively used by the masses in India for the treatment of cold and cough, whooping cough, and phlegm. It reduces inflammation of the pharynx, tonsils, and irritation or infection. The jelly like sticky mass of ripe fruit of *Cordia myxa* L. is the main ingredient, which has been reported as antiviral and antitussive (Jamkhande et al., 2013). Another important constituent is Ziziphus fruit, which contains betulinic acid. Literature showed the down-regulation of IFN-γ level by betulinic acid in mouse lung, thus enhancing immunity and suggested as potential therapeutic agent for viral infections (Hong et al., 2015). Aqueous extract also reported increasing thymus and spleen indices as well as enhance the T-lymphocyte proliferation, hemolytic activity, and natural killer (NK) cell activity (Yu et al., 2016). *Viola odorata* L*.,* one of its ingredients, suppresses the viral load (Gerlach et al., 2019). Hence, the literature supports the use of AYUSH formulation Laooq-e-Sapistan in COVID-19.

#### 3.2.6 Sharbat-e-Sadar

Sharbat-e- Sadar is an Unani polyherbal syrup formulation and is widely used for common cold, cough and respiratory diseases. *Trachyspermum ammi* (L.) Sprague*,* an important ingredient, reported to neutralize antibodies for Japanese encephalitis virus (Roy et al., 2015), and a glycoprotein was found to proliferate B-cells (Shruthi et al., 2017). *Adhatoda vasica* Nees inhibits HIV-Protease (Singh et al., 2010), *Bombyx mori* was reported to increase immune responses against viral infection (Lü et al., 2018). Other ingredients such as *Glycyrrhiza glabra* L., *Ficus carica* L., *Onosma bracteatum* Wall., and *Ziziphus jujuba* Mill. also possess the antiviral and immunomodulatory activities, as summarized in Table 5.

**TABLE 5:** List of Indian Medicinal Plants/AYUSH drugs with proven immunomodulatory, antiviral and anti-allergic/anti-inflammatory/anti-asthmatic activity having potential for exploring against COVID 19 categorized for prioritization on the basis of their earlier reports.

Category. Sl no	Botanical name/Common name/Family/Part	Immunomodulatory activity	Anti-viral activity	Anti-allergic/anti asthmatic/anti-inflammatory/respiratory disorders
C1.1	*Acacia catechu* (L.f.) Willd./Khadira/Fabaceae/Leaves, bark, heartwood	Aqueous and alcoholic extract increased phagocytic response showed by peritoneal macrophages. The extracts inhibited TNF-α and the production of NO, IL-10. **Dose: 100 and 200 mg/kg** Sunil et al. (2019)	Aqueous, hydroalcoholic and n-butanol extract showed anti HIV-1 activity by inhibiting viral protein and Tat **IC**_**50**_ **: 1.8 μg/ml** Nutan et al. (2013)	Aqueous extract of leaves showed inhibitory effects on histamine synthesis in rat peritoneal as well as mast cells. **Dose: 100 mg/kg** Prasad et al. (2009), Negi and Dave (2010)
C1.2	*Adhatoda vasica* Nees/Adusa/Acanthaceae/Leaves	Methanolic extract of leaves inhibit DTH reactiveness, increased the percentage neutrophil adhesion, promoting increased phagocytic activity vis-à-vis increased concentration of lytic enzymes for more effective killing.**Dose: 400 mg/kg** Vinothapooshan and Sundar (2011)	Ethanolic leaf extract inhibit the activity of HIV-Protease. HIV-protease plays a significant part in the replication cycle. Singh et al. (2010)	Alcoholic extract inhibited IgE-dependent basophil mediator release. **Dose: 20 mg/kg** Gibbs (2009), Hossain and Hoq (2016)
C1.3	*Aegle marmelos* (L.) Correa*/*Bael*/*Rutaceae*/*Root, stem bark, fruits	Alcoholic extract stimulates immune system by acting through cellular and humoral immunity.**Dose: 100 and 500 mg/kg** Patel and Asdaq (2010)	Purified seselin showed inhibitory potential over multiple SARS-COV-2 targets and holds a high potential to work effectively as a novel drug for COVID-19. Nivetha et al. (2020)	Aqueous extract inhibit production of nitric oxide (NO) by rat peritoneal cells, anti-histamine effect, and membrane stabilization activity. **Dose: 200 mg/kg** Kumari et al. (2014)
C1.4	*Anacyclus pyrethrum* (L.) Lag./Akkal kadha/Asteraaceae/Root	Petroleum ether extract showed cellular and humoral immunity.**Dose: 50–100 mg/kg** Sharma et al. (2010)	Pyrethrin act as ligands to bind with viral proteins to prevent the binding of host receptors preventing the fusion lead viral replication in COVID 19 Kumar et al. (2019)	-
C1.5	*Andrographis paniculata* (Burm.f.) Nees/Kalmegh*/*Acanthaceae/Leaves	Isolated compound of andrographolide modulate immune responses by regulating macrophage phenotypic polarization and MAPK and PI3K signaling pathways regulate macrophage polarization. **Dose: 10 μg/ml (*In vitro*) and 1 mg/kg (*In vivo*)** Wang et al. (2010)	Alcoholic extract inhibit the viral titer in A549 cells transfected with SRV. They showed the activity through p38 MAPK/Nrf2 pathway.**Dose: 50 μg/ml** Churiyah et al. (2015), Wintachai et al. (2015)	Andrographolide attenuate allergic asthma by inhibition of the NF-kappaB signaling pathway. **Dose: 0.1, 0.5, and 1 mg/kg** Bao et al. (2009)
C1.6	*Carica papaya* L.*/*Papaya/Caricaceae/Leaves, fruits	Alcoholic extract of fruit pulp and seed enhanced phagocytic activity of peritoneal macrophages is correlated with T helper 1 cytokine response. Interferon-gamma increases the phagocytosis process.**Dose: 0.11 g/ml extract every day using a gastric cannula** Amin et al. (2019)	Aqueous extract of the *C. papaya* leaves increases the expression of the envelope and NS1 proteins in DENV-infected THP-1 cells.**IC**_**50**_ **: 100 μg/ml** Sharma N. et al. (2019)	Alcoholic extract of leaves in mouse model of ovalbumin- (OVA) induced allergic asthma down regulates IL-4, IL-5, eotaxin, TNF-α, NF-ĸB, and iNOS levels thus exhibits anti-inflammatory effect. **Dose: 100 mg/kg** Inam et al. (2017)
C1.7	*Cassia occidentalis* L./Kasunda*/*Fabaceae/Aerial part, seeds	Isolated rhein suppresses the functional responses of the T- and B-lymphocytes and also suppresses lymphoproliferation in splenocytes.**Dose: 10 μM** Panigrahi et al. (2016)	Alcoholic extract showed that the plant possessed an anti-HIV property through inhibition of viral reverse transcriptase activity.**IC**_**50**_ **: >100 mg/ml** Estari et al. (2012)	Isolated anthraquinone showed anti-asthmatic potential by decreasing mRNA expression of Th1/Th2 cytokine in lung tissue. **Dose: 250, 500 and 2000 mg/kg** Xu et al. (2018)
C1.8	*Cocculus hirustus* (L.) Diels/Patalagarudi/Menispermaceae/Whole plant	Methanolic extract showed significantly enhanced specific and non-specific activity on various immune paradigm in cyclophosphamide induced immunosuppresed animals.**Dose: 200 mg/kg** Mallik and nayak (2014)	Found effective against all strains of dengue virus and SARS CoV 2 in *in vitro* studies, hence under phase 2 clinical trial as phytopharmaceutical drug against COVID 19 at 12 centers. (https://www.clinicaltrialsarena.com/news/sun-pharma-covid-19-trial/)	The methanolic leaf extract showed significant analgesic activity in mice as well as significant anti- inflammatory activity using *in vitro* and *in vivo* rat models. **Dose: 100 mg/kg** Sengottuvelu et al. (2012)
C1.9	*Cordia myxa* L./Sapistan/Boraginaceae/Fruits	Aqueous extract of *C. myxa* fruits significantly increased the delayed type hypersensitivity (DTH), mitotic index (MI) of bone marrow and spleen cells Ali et al. (2015)	Dichloromethane, ethyl acetate, and methanol stem extracts showed anti-viral potential against HIV-1 using the syncytia formation assay. **IC**_**50**_ **: 21.8 μg/ml** Rashed (2014)	Hydroalcoholic extract inhibit the oxidant stress factors that lead to progression of colitis.**Dose: 100 mg/kg** Ranjbar et al. (2013)
C1.10	*Curcuma longa* L./Haldi/Zingiberaceae/Rhizome	Lyophilized turmeric was found to decrease spleen weight, decrease the proportion of CD4^−^, CD8^+^ T cells, and decrease phagocytic activity. **Dose: 1 and 2% (w/w)** Kim et al. (2014). Polysaccharide fraction of aqueous extract of *C. longa* inhibiting the secretion of IL-12 and PGE2. **Dose: 0.8–500 μg/ml** Chandrasekaran et al. (2013)	Aqueous extract of *C. longa* suppressed the HBV replication and the transcription of HBV genes in HepG2 cells which produce HBV particles. **Dose: 200 mg/L and 500 mg/L** Kim et al. (2009). Isolated curcuminoids from aqueous extract of curcuma longa exhibited significant inhibitory activity against the neuraminidases from novel influenza H1N1 (WT) and oseltamivir-resistant novel H1N1 (H274Y mutant) expressed in 293 T cells. **IC**_**50**_ **: 6.18 ± 0.64 to 40.17 ± 0.79 μg/ml** Dao et al. (2012). Virtual screening of curcumin and its analogue found its activity SARS CoV 2 surface proteins and is under clinical trial. (https://chemrxiv.org/articles/Virtual screening of curcumin and its analogs against the spike surface glycoprotein of SARS-cov-2 and SARS-cov/12142383)	Alcoholic extract of *C. longa* ameliorates food allergy by maintaining Th1/Th2 immune balance in ovalbumin challenged mice. **Dose: 100 mg/kg** Shin et al. (2015)
C1.11	*Cynodon dactylon* (L.) Pers./Doorva/Poaceae/Whole plants	Fresh juice of the grass increased humoral antibody response upon antigen challenge, significant increase in antibody titer in the haemagglutination antibody assay and plaque forming cell assay. **Dose: 250 and 500 mg/kg** Mangathayaru et al. (2009)	Alcoholic dried extract showed virustatic and virucidal activity against porcine reproductive and respiratory syndrome virus (PRRSV) and also significantly inhibits replication of PRRSV. **Dose: 0.78 mg/ml** Pringproa et al. (2014)	Chloroform extract of whole plant produces a bronchodilation via antimuscarinic calcium channel blocking activators and phosphodiesterase inhibition activity. **Dose: 5, 10, 50 and 100 mg/kg** Patel et al. (2013)
C1.12	*Jatropha curcas* L./Euphorbiaceae/Leaves, roots	Phytoconstituents of hydroalcoholic extract ameliorated both cellular and humoral antibody response. **Dose: 0.25, 0.5, 1 mg/kg** Abd-Alla et al. (2009)	Successive extract of *J. curcas* was evaluated by inhibition of HIV replication as determined by HIV p24 antigen ELISA showed 100% inhibition by methanolic and 97.19% inhibition by aqueous extract. **IC**_**50**_ **: 0.0255–0.4137 mg/ml (aqueous) and 0.00073–0.1278 mg/ml (Methanolic)** Dahake et al. (2013)	Isolated jatrophacine showed anti-inflammatory potential by inhibiting production of nitric oxide in LPS-induced RAW 264.7 macrophages. **IC**_**50**_ **: 0.53 μM** Yang et al. (2019)
C1.13	*Mollugo cerviana* (L.) Ser.*/*Grishmasundara*/*Molluginaceae/Whole plant	Alcoholic extracts increase NO release by peritoneal cells. **Dose: 25 μg/ml** Ferreira et al. (2003)	Alcoholic extract exhibits antiviral properties for both chikungunya virus and dengue virus. **Dose: 1.8 mg/ml** Jain et al. (2019)	Hydroalcoholic extract inhibit the levels of lipid peroxides, acid phosphatase, and gamma-glutamyl transpeptidase activity. **Dose: 1 mg/g** Sadique et al. (1987)
C1.14	*Nigella sativa* L./Kalonji/Ranunculaceae/Seeds	Aqueous extract of *N. sativa* enhance the proliferative capacity of splenocytes and T lymphocytes, suppression of IFNγ secretion from splenocytes. **Dose: 10, 50, and 100 g/ml** Majdalawieh et al. (2010)	Nigellidine and α- hederin found to have the best potential to act as COVID-19 treatment in docking studies. (https://chemrxiv.org/articles/Identification of compounds from nigella sativa as new potential inhibitors of 2019 novel corona virus Covid-19 molecular docking study/12055716/1)*N. sativa* seeds oil possesses a striking antiviral effect against MCMV infection. **Dose: 100 mg/100 ml/mouse** Umar et al. (2016)	Aqueous extract of seed showed sensory receptors mediating reflex bronchoconstriction and tachykinin receptor antagonists. **Dose: 3.3% w/w extract** Boskabady et al. (2003)
C1.15	*Ocimum sanctum* L./Tulsi/Lamiaceae/Leaves	Aqueous extract of leaves showed regulation of IL-2 production and exhibited leukocytosis and augmentation of T& B cells. **Dose: 250 mg/kg** Goel et al. (2010)	Hydroalcoholic extract showed promising antiviral properties against H9N2 virus by inhibition of a stage in viral intracellular multiplication and non-specific interference with virus-cell interactions. **Dose: 135, 67, 33 mg/0.1 ml** Ghoke et al. (2018)Tulsinol and dihydroeugenol have been found effective against SARS CoV 2 in molecular docking studies. (https://papers.ssrn.com/sol3/papers.cfm abstract id = 3554371)	Alcoholic extracts showed anti-asthmatic potential through inflammatory mechanism by inhibiting LTC4, LTA4 and COX-2 in HL-60 cell lines and reduction in inflammation in asthma mice model. **IC**_**50**_ **: 1–10 μg/mlDose: 100 mg/kg** Soni et al. (2015)
C1.16	*Phyllanthus emblica* L.*/*Amla/Phyllanthaceae/Fruits	Alcoholic extract of fruits stimulate B and T lymphocyte and restored the interleukin production considerably. **Dose: 10 mg to 1 mg/ml** Sai Ram et al. (2002)	Fractionated alcoholic extract inhibit HIV reverse transcriptase activity. **IC**_**50**_ **: >100 mg/ml** Estari et al. (2012)	Alcoholic extract exhibits anti-inflammatory and anti-oxidant activity by protecting RAW264.7 cells from oxidative damage by increasing glutathione content and total superoxide dismutase activity, suppressing MDA content and decreasing release of pro-inflammatory mediators. **IC**_**50**_ **: 0.677 ± 0.029 mg/ml** Li W. et al. (2020)
C1.17	*Solanum nigrum* L./Makoi/Solanaceae/Seeds, barriers	Isolated polysaccharides of significant increment in the percentage of CD4^+^ T lymphocyte and a decrease in the percentage of CD8^+^ T lymphocyte of tumor-bearing mice peripheral blood. **Dose: 90, 180, 360 mg/kg** Li et al. (2009)	Chloroform extract decreased the expression or function of HCV NS3 protease in a dose dependent manner and GAPDH remained constant. **Dose: 100 μg/μL** Javed et al. (2011)	Petroleum ether extract of berries inhibits asthma by inhibiting increase in leukocyte and eosinophil count, protection against mast cell degranulation and resisting contraction due to presence of β-sitosterol. **Dose: 50, 100 and 200 mg/kg** Nirmal et al. (2012)
C1.18	*Valeriana wallichii* DC./Valerianaceae/Roots	Alcoholic root extract inhibited HCV by binding with HCV NS5B protein.**Dose: 250 μg/ml** Ganta et al. (2017)	Alcoholic extract and its fraction inhibit HCV by binding with HCV NS5B protein. **Dose: 200 μg/ml** Ganta et al. (2017)	Crude extract showed protection against airway disorders through relax ion of the low K^+^ (25 mM)-induced contractions with a mild effect on the contractions induced by high K^+^ (80 mM). **Dose: 0.03–3.0 mg/ml** Khan and Gilani (2012)
C1.19	*Vitex negundo* L./Renuka/Verbanaceae/Leaves	Hydroalcoholic extract of leaves of *V. negundo* activate the phagocytic cells such as macrophages and neutrophils. **Dose: 200 mg/kg** Lad et al. (2016)	Alcoholic extract of leaves inhibits HIV-1 reverse transcriptase activity in *in vitro* assay thus exhibits anti-HIV activity. **Dose: 200 μg/ml** Kannan et al. (2012)	*V. negundo* leaf oil inhibit COX-2 without much interfering COX-1 pathways. **Dose: 500 μL/kg** Chattopadhyay et al. (2012)
C1.20	*Withania somnifera* (L.) Dunal/Asgand/Solanaceae/Roots	Aqueous suspension of root showed potent inhibitory activity toward mitogen induced proliferative response of T-lymphocyte and delayed-type hypersensitivity reaction. **Dose: 1000 mg/kg** Rasool and Varalakshmi (2006)	Hydro-alcoholic root extract of *W. somnifera* showed antiviral properties against IBD virus by cytopathic effect reduction assay. **Dose: 25 μg/ml** Pant et al. (2012). Withanone and withaferin a have been found effective against SARS CoV 2 in bioinformatics studies and asgandh extract is under clinical trial. (https://www.researchsquare.com/article/rs-17806/v1), (http://www.bioinformation.net/016/97320630016411.pdf)	Aqueous extract of withania root inhibit histamine and 5-HT in early phase and prostaglandins in delayed phase of inflammatory reaction. **Dose: 1000 mg/kg** Sahni and Srivastava (1993)
C1.21	*Zingiber officinale* Roscoe/Sunthi/Zingiberaceae/Rhizome	Volatile oil of ginger influences both cell-mediated immune response and nonspecific proliferation of T lymphocyte. **Dose: 0.125, 0.25, and 0.5 g/kg** Zhou et al. (2006)	Aqueous extract effective against HRSV-induced plaque formation on airway epithelium by blocking viral attachment and internalization. **IC**_**50**_ **: >150 μg/ml** Chang et al. (2013)	Aqueous and alcoholic extract showed anti-asthmatic effect by reducung inflammation through suppression of Th2-mediated immune response. **Dose: 500 mg/kg (alcoholic extract)720 mg/kg (aqueous extract)** Khan et al. (2015)
C2.1	*Abutilon indicum* (L.) Sweet/Tuthi/Malvaceae/Aerial parts	Alcoholic extract showed stimulatory effect on T lymphocytes. Increasing doses of showed higher HA titer value, restoration of WBC count. It also increased lymphocyte and E-rosette formation. **Dose: 200 and 400 mg/kg** Gaikwad and Krishna Mohan (2012)	Alcoholic extract of leaves showed anti-MCV and anti-HSV activities. **Dose: 0.4 μg/ml** Vimalanathan et al. (2009)	Methanolic extract of aerial part showed mast cell stabilizing and anti-inflammatory activity. **Dose: 250 and 500 mg/kg** Mehta and Paranjape (2008)
C2.2	*Achyranthes aspera* L.*/*Apamarga*/*Amaranthaceae*/*Root	Polyphenolic compounds of hydroalcoholic extract showed cytokine based immunomodulatory role. **Dose: 100 mg/kg** Narayan and Kumar (2014)	Alcoholic extract showed potential activity against herpes simplex virus type-1 and type-2 by inhibiting the early stage of multiplication in vero cells. Mukherjee et al. (2013)	Ethyl acetate fraction from methanolic extract showed *in vitro* anti-inflammatory activity. **IC**_**50**_ **: 50 = 76 ± 0.14** Khuda et al. (2013)
C2.3	*Aloe vera* (L.) Burm.f*.*/Ghrit kumari*/*Asphodelaceae/Roots, leaves	Aloe vera gel administration did not increase ovalbumin (OVA)- specific cytotoxic T lymphocyte (CTL) generation in normal mice. **Dose: 100 mg/kg** Im et al. (2010)	Isolated anthraquinone showed anti-viral activity by inhibiting virus replication. **IC**_**50**_ **: 13.70 ± 3.80 to 62.31 ± 3.05** Borges-Argáez et al. (2019)	Polysaccharide isolated from gel showed anti-allergy potential by inhibition of type 2 helper T cell (Th_2_) immune response, increase in IL-10 production and stimulating type 1 regulatory T (Tr1) cells activation. **Dose: 50 and 100 mg/kg** Lee D. et al. (2018)
C2.4	*Alstonia scholaris* (L.) R.Br.*/*Saptaparni*/*Apocynaceae/Bark	Aqueous extract enhanced phagocytic activity. **Dose: 50 mg/kg** Iwo et al. (2000)	Aqueous and alcoholic plant extract showed anti-viral potential against coxsackie B2, polio virus and herpes simplex virus. **Dose: 2.8 mg/kg** Antony et al. (2014)	Alcoholic extract inhibited inflammatory response by through reduction in ovalbumin-provoked airways allergic inflammatory stress. **Dose: 10, 25, and 50 mg/kg** Zhao et al. (2017)
C2.5	*Azadirachta indica* A.Juss./Neem/Meliaceae/Leaves	Dried powdered leaves significantly enhanced the antibody titers against new castle disease virus (NCDV) antigen. **Dose: 2 g/kg** Sadekar et al. (1998)	Isolated polysaccharides from aqueous extract of the leaf virucidal aginst Poliovirus-1 (inhibiting initial stage of viral replication). **IC**_**50**_ **: 80 μg/ml and 77.5 μg/ml** Faccin-Galhardi et al. (2012)	Aqueous leaves extract showed anti-inflammatory and analgesic activity by in chemical and thermal induced pain models in albino rats. **Dose: 500 mg/kg** Buchineni et al. (2014)
C2.6	*Berberis aristata* DC./Daruharidra/Berberidaceae/Bark	Isolated berberine inhibited the suppressed viral infection-induced up-regulation of TLR7 signaling pathway. **Dose: 20 mg/kg** Yan et al. (2018)	Isolated compound of berberine inhibited EV71 replication by down regulating autophagy and MEK/ERK signaling pathway. **IC**_**50**_ **: 7.43 to 10.25 μM** Wang et al. (2017)	Hydroalcoholic extract showed anti-inflammatory potential, which may be attributed to its inhibitory activity on macrophage-derived cytokine and mediators. **Dose: 50, 100, and 200 mg/kg** Kumar et al. (2016)
C2.7	*Bergenia ciliata* (Haw.) Sternb.*/*Pashanbheda/Saxifragaceae/Stem	Alcoholic extract stimulated the expression of CD69 on lymphocytes. **Dose: 3.13 and 6.25 mg/ml** Tumova et al. (2018)	Alcoholic extract showed potent anti-viral activity against both influenza virus a and HSV-1. **IC**_**50**_ **: >6.25 μg/ml** Rajbhandari et al. (2009)	Alcoholic extract exhibited significant anti-inflammatory activity in carrageenan-induced rat paw oedema manner. **Dose: 300 mg/kg** Sinha et al. (2001)
C2.8	*Camellia sinensis* (L.) Kuntze/Chary/Thecae/Leaves	Aqueous extract of *C. sinensis* changes hematological profile, immuno potentiating cells, cellular response in splenectomised mice. **Dose: 250 and 500 mg/kg** Gomes et al. (2014)	Hydroalcoholic extract of *C. sinensis* inhibited ADV replication in post-adsorption stage. **IC**_**50**_ **: 6.62 μg/ml** Karimi et al. (2016)	Aqueous extract showed anti-asthmatic potential by increasing expression of Th1 cell-specific anti-asthmatic biomarkers (tumor necrosis factor-ß and interferon-γ) and decreasing the expression of anti-asthmatic cytokines in the lungs. **Dose: 25 μg/ml** Heo et al. (2008)
C2.9	*Cannabis sativa* L.*/*Vijaya/Cannabaceae/Leaves	Cannabinoids indicating mainly immunosuppressive effects on macrophages, NK cells, T lymphocytes and their ability to produce cytokines. **Dose: 5 mg/day** Killestein et al. (2003)	*C. sativa* inhibited viral DNA synthesis. It inhibit the replication cycle of various types of DNA or RNA viruses Jassim and Naji (2003) Hot water extract *C. sativa* reduced the plaque forming ability. **Dose: 300–500 μg/ml** Kurokawa et al. (1993)	Oil extract of *C. sativa* protective effect against COPD through affecting the expression of specific airway epithelial cell genes that modulate Th1 processes using *in vitro* assay. **Dose: 2.4, 1.2, and 0.6 μg/ml** Mamber et al. (2020)
C2.10	*Cassia fistula* L./Amaltas/Fabaceae/Bark	Hydro alcoholic extract of *C. fistula* increased antibody titer against salmonella typhimurium ‘O’ antigen and significant enhancement in skin thickness in DNCB sensitized albino rats. **Dose: 125 mg/kg, 250 mg/kg and 500 mg/kg** Laxmi (2015)	Hydroalcoholic extract of *C. fistula* suppressed extracellular HBV DNA production. **Dose: 100 μg/ml** Indrasetiawan et al. (2019)	Isolated rhein showed anti-inflammatory activity by modulating levels carrageenan-induced hind paw edema, croton oil-induced ear oedema, cotton pellet-induced granuloma and acetic acid-induced vascular permeability models. **Dose: 10 mg/kg** Antonisamy et al. (2019)
C2.11	*Cinnamomum verum* J.Presl./Daarchini/Lauracea/Stem, bark	Bark suspension increased the phagocytic index in carbon clearance test, neutrophil adhesion and serum immunoglobulin levels and antibody titer values. **Dose: 10 and 100 mg/kg** Niphade et al. (2009)	Aqueous extract provide treatment against influenza virus infections in vero cells transfected with H7N3 influenza. Brochot et al. (2017)	Isolated procyanidine showed reduction in the elevated levels of total protein, albumin, goblet cell hyperplasia and inflammatory cell infiltration in lung tissue. **Dose: 10, 30, and 100 mg/kg** Kandhare et al. (2013)
C2.12	*Cissampelos pareira* L./Akamai/Menispermaceae/Aerial parts, roots	Isolated alkaloid fraction of alcoholic extract modulate both T and B cell mediated immune response. **Dose: 100 mg/kg** Bafna and Mishra (2010)	Alcoholic extract of aerial part of *C. pareira* inhibit the viral replication and ability to down-regulate the production of TNF-α, a cytokine implicated in severe dengue disease. **IC**_**50**_ **: ≥125 μg/ml** Sood et al. (2015)	Alkaloids fraction suppressed the production of nitric oxide, a critical mediator in inflammation. **Dose: 100 mg/kg** Bafna and Mishra (2010)
C2.13	*Cyperus rotundus* L./Musta/Cyperaceae/Rhizome	Aqueous, alcoholic, ethyl acetate and total oligomer flavonoids (TOF) extracts of *C. rotundus* influence humoral-mediated immunity by stimulating B and T cell proliferation. **Dose: 1–1000 μg/ml** Soumaya et al. (2013)	Aqueous, alcoholic and ethyl acetate extract of *C. rotundus* inhibited the HBV DNA replication in HepG2.2.15 cell line. **IC**_**50**_ **: 29.0, 21.5, 263.4** Xu et al. (2015)	Isolated sesquiterpenes from alcoholic extract showed anti-allergic potential against immediate-type as well as delayed-type hypersensitivity. **Dose: 300 μg/ml (*in vitro*) and 50–300 mg/kg (*in vivo*)** Jin et al. (2011)
C2.14	*Daphne gnidium* L./Lota/Thymelaeaceae/Aerial part	Dichloromethane extract of the aerial exhibited strong antiretroviral activity by interference with HIV co-receptors, CCR5 and CXCR4. Vidal et al. (2012)	Dichloromethane extract of the aerial parts exhibited strong antiretroviral activity and absence of cytotoxicity and pure compounds were active against multidrug-resistant viruses irrespective of their cellular tropism. **Dose: 10 μg/ml** Vidal et al. (2012)	Ethyl acetate extract showed anti-inflammatory effects by inhibiting macrophage proinflammatory function by reducing LPS-induced production of IL-1β, TNF-α, COX-2-derived PGE2 and iNOS-II-synthesized NO. **Dose: 1–100 μg/ml** Harizi et al. (2011)
C2.15	*Ficus carica* L./Anjeer*/*Moraceae/Leaves, latex	Administration of extract ameliorated both cellular and humoral antibody response Patil et al. (2010)	Resuspension of latex in DMEM containing 1% ethanol able to interfere with the replication of CpHV-1. **IC**_**50**_ **: 100 μg/ml** Camero et al. (2014)	Tea infusion of leaves showed anti-allergy potential through promotion of dissociation of IgE from FcεRI receptors. **Dose: 10 ml/kg** Abe (2020)
C2.16	*Glycyrrhiza glabra* L*./*Mulethi/Fabaceae*/*Roots, rhizome and leaves	Aqueous root extract showed leukocyte count and phagocytic index increased as well as cellular immune response study, an enhancement in foot pad thickness was observed. **Dose: 1.5 g/kg** Mitra Mazumder et al. (2012)	Aqueous and alcoholic extracts of *G. glabra* verified hemagglutination (HA) test data through which amount of virus is quantified from the allantoic fluid of chicken embryos. **Dose: 300 μg/ml** Ashraf et al. (2017)	Saponin fraction showed anti-asthmatic potential in triple antigen sensitized rats by inhibition of mast cell degranulation. **Dose: 100 mg/kg** Patel et al. (2009)
C2.17	*Illicium verum* Hook.f./Takkola*/*Magnoliaceae/Fruit	Isolated lectins from *I. verum* showed immunomodulatory action by stimulating phagocytic function. **Dose: 30 and 50 mg/kg** Bouadi et al. (2015)	Aqueous, alcoholic and hydroalcoholic extracts exhibited inhibitory effects against NDV and avian reovirus. **Dose: 0.24–3.9 mg/ml** Alhajj et al. (2020)	70% alcoholic extract exert antiasthmatic effects through upregulation of Foxp3^+^ regulatory T cells and inhibition of Th2 cytokines. **Dose: 50, 100, and 200 mg/kg** Sung et al. (2017)
C2.18	*Mentha × piperita* L*./*Peppermint/Lamiaceae/Leaves	Hydrodistillate fractions of *M. piperita* affect the functional responses of human PMNs and PBMCs. **Dose: 2 mM and 12 µL** Cosentino et al. (2009)	Alcoholic extract showed antiviral activity against RSV with a high selectivity index, and significantly decreased the production of NO, TNF-α, IL-6, and PGE2 in lipopolysaccharide-stimulated RAW 264.7 cells. **IC**_**50**_ **: 10.41 μg/ml** Li et al. (2017)	Essential oil showed antispasmodic activity by regulating prostaglandins and nitric oxide synthase on rat trachea. **Dose: 1–300 μg/ml** de Sousa et al. (2010)
C2.19	*Mentha spicata* L./Spearmint/Lamiaceae/Leaves	Essential oil from *M. spicata* proliferate T-cells, IL-2 and potently inhibit the production of pro-inflammatory cytokine TNF-α production Orhan et al. (2016)	Aqueous extract exhibits anti-viral potential against porcine parvovirus (PPV) *in vitro* by efficiently killing them and control their multiplication in cells. **IC**_**50**_ **: 0.0340 mg/ml** Weili et al. (2011)	Ethyl acetate soluble fraction of leaves by inhibit antigen stimulated rat basophile. Prasad et al. (2009)
C2.20	*Momordica charantia* L./Bitter guard/Cucurbitaceae/Leaves, fruits and seed	Alcohol and diethyl ether extract has been found that the exposure of neutrophils and macrophages stimulates both their capacity to ingest foreign particles and their intracellular killing activities*.* **Dose: 250, 500, 1000 mg/kg** Mahamat et al. (2020)	Crude protein fraction of *M. charantia* strongly inhibited H1N1, H3N2 and H5N1 subtypes. **IC**_**50**_ **: 40–200 𝜇g/ml** Pongthanapisith et al. (2013)	Alcoholic extract showed the highest reduction of LPS-induced NO, iNOS and prostaglandin E2 production and down regulates pro-interleukin-1β and NF- ĸB activation expression in RAW 264.7 macrophages Lii et al. (2009)
C2.21	*Morus alba* L./Sahatoot/Moraceae/Leaves, fruits	Isolated water soluble polysaccharides stimulates murine RAW264.7 macrophage cells to release chemokines and proinflammatory cytokines. Lee et al. (2013). Alcoholic extract of leaves significant increase in the phagocytic index and adhesion of neutrophils. **Dose: 100 mg/kg and 1 g/kg** Bharani et al. (2010)	*M. alba* fruits juice and its fractions inhibit internalization and replication of MNV-1, whereas it may influence adherence or internalization of FCV-F9 virions. **EC**_**50**_ **: 0.005 (MNV-1) and 0.25–0.30 (FCV-F9)** Lee et al. (2014)	Juice of *M. alba* fruits inhibit production of NO and proinflammatory cytokines (TNF-α, IL-6), as well as the expression of NOS2 and PTGS2 in LPS-stimulated RAW264.7 macrophages. **Dose: 0.1, 0.5, and 1 μg/ml** Jung et al. (2019)
C2.22	*Nyctanthes arbor-tristis* L.*/*Parijata/Oleaceae/Leaves, flowers and seeds	Immunostimulant activity of NAFE seems to be mediated through splenocytes proliferation and increased production of cytokines, especially IL-2 and IL-6 of aqueous extract of Nyctanthes arbor-tristis. **Dose: 400 and 800 mg/kg** Bharshiv et al. (2016)	n-Butanol fraction of alcoholic extract of protected encephalomyocarditis virus (EMCV) infected mice against semliki forest virus (SFV). **Dose: 125 mg/kg** Gupta et al. (2005)	Alcoholic extract showed anti-asthmatic and anti-tussive activity against histamine and acetylcholine cocktail induced asthma and citric acid induce cough in Guinea pig. **Dose: 100, 200, and 300 mg/kg** Mathur et al. (2016). Extracted polysaccharide from leaves aqueous extract reduce the number of cough efforts without influencing the specific airway resistance, it triggers cough reflex provocation. **Dose: 25 and 50 mg/kg** Ghosh et al. (2015)
C2.23	*Ocimum basilicum* L./Basil/Lamiaceae/Leaves	Hydroalcoholic extract of leaves increased the IFN-γ/IL-4 ratio and decreasing BALF levels of IgE, PLA_2_ and TP. **Dose: 50,300, 600 mg/kg** Eftekhar et al. (2019b)	Alcoholic extract inhibit ZIKV replication in vero E6 cells. The extract seems to inhibit the virus at the step of attachment and entry into the host cell. **IC**_**50**_ **: 1:134** Singh et al. (2019)	Hydroalcoholic extract showed therapeutic effect on asthma by reducing eosinophil’s, monocytes, neutrophils percentage and increase in percentage of lymphocytes and antioxidant biomarkers levels. **Dose: 0.75, 1.50, and 3.00 mg/ml** Eftekhar et al. (2019a)
C2.24	*Oleo europea* L./Zaitoon/Oleaceae/Leaves	Isolated oleuropein from hydroalcoholic extract showed lymphocyte activation and proliferation properties. Oleuropein exhibited a high degree of lymphocyte aggregation. **Dose: 540 μg/ml** Randon and Attard (2007)	Aqueous leaves extract showed anti-viral potential against newcastle disease virus by restricting replication. **Concentration: 1000 μg/ml** Salih et al. (2017)	Essential oil from leaves inhibit NFB activation in monocytes and monocyte derived macrophages. Lucas et al. (2011)
C2.25	*Panax ginseng* C.A.Mey./Ginseng/Araliaceae/Roots	Ginsenosides increased the number of spleen plaque-forming cells, the titers of sera hemagglutinins as well as the number of antigen-reactive T-cells and splenocyte natural killer activity. **Dose: 10 mg/kg** Buriana et al. (1990)	Fermented extract improved the survival of human lung epithelial cells, inhibits RSV replication, suppressed the expression of RSV-induced inflammatory cytokine genes and the formation of ROS in epithelial cell cultures. **Dose: 25 mg/kg** Wang et al. (2018)	*P. ginseng* extract showed anti-asthmatic activity by restoring EMBP(eosinophil major basic protein), Muc5ac, CD40, and CD40L expression and mRNA and protein levels of IL-1, IL-4, IL-5, and TNF-α. **Dose: 20 mg/kg** Kim and Yang (2011)
C2.26	*Peganum harmala* L./Harmal/Nitrariaceae/Aerial parts and seeds	Alcoholic extract (80%) of seed showed effects on zymosan-A activated neutrophils (PMNs). **Dose: 25, 50, and 100 μg/ml** Koko et al. (2008)	Alcoholic extract inhibits viral RNA replication and viral polymerase activity. **IC**_**50**_ **: 9.87 μg/ml** Moradi et al. (2017)	Alkaloid fraction of alcoholic extract showed potent antitussive, expectorant and bronchodilating activities in cough models of mice and Guinea pigs. **Dose: Total extract (1650 mg/kg) and alkaloid fraction (90 mg/kg)** Liu et al. (2015)
C2.27	*Phyllanthus amarus* Schumach. and Thonn./Bhui amla/Phyllanthaceae/Whole plant	Alcoholic extract of aerial parts exhibited potent inhibitory action on both phagocytic and CD18 expression of phagocytes. **Dose: 6.25–100 μg/ml** Jantan et al. (2014)	Aqueous extract inhibited cellular proliferation and suppressed HBsAg production in human hepatoma cells. **Dose: 1 mg/ml** Yeh et al. (1993)	Alcoholic extract attenuates asthma by modulating oxido-nitrosative stress SOD, GSH, MDA, and NO), immune-inflammatory makers (HO-1, TNF-α, IL-1β, and TGF-β1), and Th2 cytokines. **Dose: 100 and 200 mg/kg** Wu et al. (2019)
C2.28	*Picrorhiza kurroa* Royle ex Benth.*/*Kutki/Plantaginaceae/Rhizome, leaves	Hydroalcoholic extract stimulate cell-mediated and humoral immunities, along with complement activity and phagocytic function*.* **Dose: 25, 50, 100 mg/kg** Sharma et al. (1994)	Isolated iridoids from chloroform fractionated alcoholic extract of inhibit expression of vpr in TREx-HeLa-vpr cells and these iridoid are naturally occurring vpr inhibitors. **Dose: 5 and 10 μg/ml** Win et al. (2019)	Alcoholic extract showed anti-asthmatic potential by exhibiting relaxation effect against histamine and acetylcholine induced contraction model in Guinea pigs.**Dose: 25 mg/kg (*in vivo*)1, 10 and 100 mg/ml (*in vitro*)** Sehgal et al. (2013)
C2.29	*Piper longum* L./Pipli/Piperaceae/Fruits	Aqueous extract possessed a demonstrable immunostimulatory activity, both specific and nonspecific, as evident from the standard test parameters such as haemagglutination titer, macrophage migration index and phagocytic index. **Dose: 225 mg/kg** Tripathi et al. (1999)	Butanol fraction of alcoholic extract possessed remarkable inhibitory HBV activity, against the secretion of hepatitis B virus surface antigen (HBsAg) and hepatitis B virus e antigen (HBeAg). **IC**_**50**_ **: 0.15 mM for HBsAg and 0.14 mM for HBeAg** Jiang et al. (2013)	Aqueous and pet ether extract showed anti-asthmatic potential by protecting against histamine induced bronchospasm, haloperidol induced catalepsy and passive paw anaphylaxis and by decreasing number of leukocytes in milk-induce leukocytes model. **Dose: 50, 100, and 200 mg/kg** Kaushik et al. (2012)
C2.30	*Piper nigrum* L./Marica/Piperaceae/Fruits	Aqueous extract of *P. nigrum* capable of promoting the proliferative signaling pathways in splenocytes and enhance murine splenocyte proliferation. **Dose: 50 and 100 *μ*g/ml** Majdalawieh and Carr (2010)	Isolated piperamides from *P. nigrum *inhibit coxsackie virus type B3 (CVB3). It inhibit the proliferation of VSMCs. **IC**_**50**_ **: 21.6µM to10.6 μg/ml** Mair et al. (2016)	Isolated piperine acted partially through stimulation of pituitary adrenal axis. **Dose: 5, 10, 20, and 40 mg/kg** Tasleem et al. (2014)
C2.31	*Pongamia pinnata* (L.) Pierre/Karanj/Fabaceae/Seeds	Isolated oil impact on immune cell signaling events needed for continued recruitment of neutrophils/other cells. **Dose: 0.3 or 0.5 g/kg** Muniandy et al. (2018)	Aqueous extract interfered with HBsAg and thus probably may prevent HBV entry. **Dose: 5 mg for 0.18 pg/ml concentrations of the virus** Mathayan et al. (2019)	Isolated isoflavone and showed inhibitory effects against NO production in LPS-stimulated BV-2 microglial cell thus anti-inflammatory effects. **IC**_**50**_ **: 9.0 µM** Wen et al. (2018)
C2.32	*Punica granatum* L./Anar*/*Punicaceae/Fruit, peel	Aqueous extract showed, a significant decrease in nitric oxide levels and TNF-α levels. A significant diminution of iNOS, TNF-α and NF-κB expression was also observed. **Dose: 0.65 g/kg** Labsi et al. (2016)	Alcoholic extract inhibited influenza a PR8 virus replication in the MDCK cell line, it could suppress the amplification of the infectious influenza viruses. **IC**_**50**_ **: 6.45 μg/ml** Moradi et al. (2019)	Isolated galloyl-hexahydroxydiphenoyl-glucose showed protective effect against acute lung injury and anti-inflammatory activity by inhibiting LPS-induced JNK and NF-κB activation and reduction in expression of the TNF-α, IL-6, and IL-1β genes in lungs. **Dose: 5, 50, and 100 mg/kg** Pinheiro et al. (2019)
C2.33	*Plantago major* L./Lahuriya/Plantaginaceae/Whole plants, seeds	Aqueous extract increased lymphocyte proliferation and secretion of interferon-γ at low concentrations (<50 μg/ml), but at high concentrations, it can inhibit this property (<50 μg/ml). **Dose: 50 μg/ml** Chiang et al. (2003)	Isolated compound of caffeic acid from aqueous extract possesses interesting anti-HSV-1, anti-HSV-2 and anti-ADV-3 activities. Caffeic acid was found to inhibit HSV-1 replication. **EC**_**50**_ **: 15.3 μg/ml** Chiang et al. (2002)	Hydroalcoholic extract showed amelioration of asthma by increasing mean mast cells, alveolar epithelium thickness and glycoprotein accumulation. **Dose: 100 mg/kg** Farokhi and Khaneshi (2013)
C2.34	*Psoralea corylifolia* L./Babchi/Fabaceae/Seeds	Hydroalcoholic extract stimulate natural killer cell activity. A positive response was also observed in the ADCC activity of spleen cells. **Dose: 100 and 200 mg/kg** Latha et al. (2000)	Aqueous extract found more effective in suppressing the virosis and reduced the mortality against virosis cellular and biochemical changes. Kiran Kumar et al. (2012)	Extract showed novel agent for asthma by inhibiting eosinophils accumulation into airways and modulating Th1/Th2 cytokine balance. **Dose: 200 and 400 mg/kg** Lee and Kim (2008), Wen et al. (2018)
C2.35	*Rhodiola rosea* L./Rhodora/Crassulaceae/Whole plant	Isolated compound of could promote the activation of T lymphocytes, differentiate them into CD4^+^ cell or CD8^+^ cell, and implement their functions. **Dose: 12.5, 25, 50 μg** Guan et al. (2011)	Alcoholic extract inhibit the entry and infection of ebola and marburg viruses. **IC**_**50**_ **: 0.25 μg/ml (ebola virus)4.0 μg/ml (marburg virus)** Cui et al. (2018)	Isolated salidroside showed protective effect in acute lung injury by decrease in the *W*/*D* ratio, myeloperoxidase activity of lung, reducing protein concentration, macrophages in the bronchoalveolar lavage fluid and regulating inflammatory cytokines and NF-κB. **Dose: 120 mg/kg** Guan et al. (2012)
C2.36	*Santalum album* L.*/*Sandalwood*/*Santalaceae/Stem	Aqueous extract inhibited cell proliferation, nitric oxide production and CD14 monocyte. **Dose: 30 mg/ml** Gupta and Chaphalkar (2016)	β-Santalol from hexane extract exhibits anti-influenza A/HK (H3N2) virus by inhibition of viral mRNA synthesis. **Dose: 100 μg/ml** Paulpandi et al. (2012)	Alcoholic extract showed *in vitro* anti-inflammatory activity as compared to Diclofenac. **Dose: 500 mg/ml** Saneja et al. (2009)
C2.37	*Saussurea lappa* (Decne.) C.B.Clarke/Kutha/Compositae/Roots	Isolated compound of costunolide and dehydrocostus lactone showed suppressive effect on the expression of the hepatitis B surface antigen (HBsAg) in Hep3B cells. **IC**_**50**_ **: 1.0–2.0 μM** Chen et al. (1995)	Hexane fraction of alcoholic extract suppress the HBsAg production by Hep3B cells. **IC**_**50**_ **: 1.0–2.0 µM** Chen et al. (1995)	SML0417, epiligulyl oxide and elecampane camphor isolated from roots ameliorates allergic asthma in murine model by inhibiting antigen-induced degranulation, reduction in inflammatory signs and mucin production and expression and secretion of Th2 cytokines. Lee B. K. et al. (2018)
C2.38	*Sphaeranthus indicus* L./Mundi/Asteriae/Leaves, flowers	Petroleum ether extract from the flower heads of *S. indicus* increasing phagocytic activity, hemagglutination antibody titer and delayed type hypersensitivity. **Dose: 200 mg/kg** Bafna and Mishra (2007)	Alcoholic extract exhibits anti-virus potential against herpes simplex virus (HSV) and mouse corona. **Dose: 0.4 μg/ml** Vimalanathan et al. (2009)	Alcoholic leaves extract inhibit prostaglandin synthesis. **Dose: 100,200, and 400 mg/kg** Meher et al. (2011)
C2.39	*Syzygium aromaticum* (L.) Merr. and L.M. Perry*/*Lavang*/*Myrtaceae/Fruits	Aqueous and alcoholic suppressive effects on mouse macrophages and inhibit IL-1*β*, IL-6, and IL-10. **Dose: 1000 μg/ml** Dibazar et al. (2015). Essential oil increased the WBC count and enhanced DTH response in mice. Carrasco et al. (2009)	Hydroalcoholic extract exhibits anti- viral activity against herpes simplex virus-1 evaluated on vero cell line using MTT assay. **IC**_**50**_ **: 8.4 μg/ml** Moradi et al. (2018)	Aqueous extract decreases neutrophil count and proteins leakage into bronchoalveolar lavage fluid. **Dose: 200 mg/kg** Chniguir et al. (2019)
C2.40	*Terminalia chebula* Retz.*/*Halala/Combretaceae/Fruits	Aqueous extract increase in humoral antibody titer and delayed-type hypersensitivity in mice. **Dose: 100–500 mg/kg** Shivaprasad et al. (2006)	Hydroalcoholic extract of prevents the attachment as well as penetration of the HSV-2 to vero cells and efficacy to inhibit virus attachment and penetration to the host cells. **IC**_**50**_ **: 0.01 ± 0.0002 μg/ml** Kesharwani et al. (2017)	Carbohydrate polymer from aqueous extract of dried ripe fruit showed antitussive efficacy in citric acid-induced cough efforts. **Dose: 50 mg/kg** Nosalova et al. (2013). Ethyl acetate fraction showed antitussive efficacy on sulfur dioxide gas induced cough partially through modulation of opioid receptors. **Dose: 500 mg/kg** Haq et al. (2013)
C2.41	*Tinospora cordifolia* (Willd.) Miers/Giloe/Menispermaceae/Stem	Aqueous and alcoholic extract reduced bacterial load as compared to untreated macrophages. **Dose: 100, 200, and 500 μg/ml** Alsuhaibani and Khan (2017)	Methanol and ethyl acetate mixture extract inhibits the growth of HSV. **Dose: 50–100 μg/ml** Pruthvish and Gopinatha (2018)	Hydroalcoholic extract ameliorates asthma through decreasing oxidative stress and inflammation through modulating glutathione homeostasis and regulation of NFκB and pro-inflammatory genes. **Dose: 100 mg/kg** Tiwari et al. (2014)
C2.42	*Tribulus terrestis* L./Gokhru*/*Zygophyllaceae*/*Whole plant	Saponin fraction increased phagocytic activity in dose dependent manner. **Dose: 50, 100, 200 μg/ml** Tilwari et al. (2011)	Alcoholic extract showed antiviral potential against newcastle disease virus evaluated by titering *in vivo* vero cell line culture. **Dose: 80 μg/ml** Malik et al. (2018)	Hydroalcoholic fruit extract activate mast cell. **EC**_**50**_ **: 1% extract with 0.1% HC** Kang et al. (2017)
C2.43	*Ziziphus jujuba* Mill./Unnab/Rhamnaceae/Fruits	Aqueous extract increase thymus and spleen indices as well as enhance the T-lymphocyte proliferation, hemolytic activity and NK cell activity. **Dose: 1.3, 2.6, and 5.2 g/kg** Yu et al. (2016)	Isolated betulinic acid showed antiviral activity on influenza virus by attenuating pulmonary pathology and down-regulation of IFN-γ level. **Concentration: 50 μM** Hong et al. (2015)	Alcoholic extract showed inhibition of expression and activity of COX-2. **Dose: 200, 400, and 600 mg/kg** Mesaik et al. (2018)
C2.44	*Zataria multiflora* Boiss./Satar/Lamiaceae/Whole plant, leaves	Obtained essential oils from hydrodistillation increase in the secretion of TNF-α, IFN-γ, IL-2 and decrease in IL-4. **Dose: 10mg/one BALB/c and 7mg/one C57BL/6** Jamali et al. (2020)	*Z. multiflora* destruction of virus infectivity or inhibition of early phases of viral proliferation cycle. Arabzadeh et al. (2013)	Hydro-alcoholic extract ameliorates allergic asthma by decreasing pro-inflammatory cytokines, increasing expression of anti-inflammatory cytokines gene and number of treg (FOXP3) in splenocytes. **Dose: 200, 400, and 800 μg/ml** Kianmehr et al. (2017)
C3.1	*Artemisia absinthium* L./Vilayati afsantin/Asteraceae/Roots	Alcoholic extract modulates the percentage expression and fluorescent intensity of CD86, CD40 and MHC II molecules on DCs. **Dose: 100 μg/ml** Azeguli et al. (2018)	Decoction effectively suppressed HBV DNA, HBeAg, and HBsAg.**Dose: 15 ml (containing 1 g of dried extract**) Ansari et al. (2018)	-
C3.2	*Datura metel* L./Safed dhatura/Solanaceae/Leaves, fruits and seeds	-	Aqueous and alcoholic extract performed in vero cell line using MTT assay showed good antiviral activity. **IC**_**50**_ **: 2.5 mg/ml** Roy et al. (2016)	Aqueous extract in ovalbumin challenged mice ameliorates asthma through promotion of naïve T cell development and reducing activated T cells. **Dose: 0.56 mg/kg** Rifa’i et al. (2014)
C3.3	*Elettaria cardamomum* (L.) Maton/Chotielaichi/Zingiberaceae/Fruits	Essential oil overlapped with that of various canonical signaling pathways which support its immunomodulator activity. Han and Parker (2017)	-	The extract obtained from supercritical fluid extraction with carbon dioxide inhibit NF-kappa signaling pathway. **Dose: 0.03%** Souissi et al. (2020)
C3.4	*Embelia* ribes Burm.f./Baberang/Myrsinacea/Fruits	-	Ethyl acetate extract exhibits antiviral activity MDCK cells infected with influenza virus A/Puerto rico/8/34 (H1N1). **IC**_**50**_ **: 0.2 μg/ml** Hossan et al. (2018)	Isolated embelin attenuates anti-inflammatory activity against carrageenan induced paw edema in rats. **Dose: 20 mg/kg** Mahendran et al. (2011)
C3.5	*Hedychium spicatum* Sm.*/*Kapurkachri*/*Zingiberaceae/Rhizome	Alcoholic extract increased phagocytosis, WBC and neutrophils count. **Dose: 200–500 mg/kg** Uttara and Mishra (2009)	-	Aqueous extract attenuates anti-histaminic action against histamine-induced bronchospasm in Guinea pig. **Dose: 200 mg/kg** Ghildiyal et al. (2012)
C3.6	*Hyssopus officinalis* L./Zoofa/Lamiaceae/Flowers, leaves	Alcoholic extract of leaves inhibits plaque formation of both of the two strains of HSV-1 in vero E6 cells. **Dose: 125 mg/kg** Behbahani (2009)	Aqueous extract of flowers affect the levels of some cytokines (such as IL-4, IL-6, IL-17, and IFN-γ) in asthmatic mice. By detection of the expressions of MMP-9 and TIMP-1 and the morphological changes. **Dose: 0.04 g/10 g** Ma et al. (2014)
C3.7	*Inula racemosa* Hook.f./Puskara/Asteraceae/Root	Polysaccharide fraction of water extract showed immunomodulatory action by stimulating phagocytic function. **Dose: 100–200 mg/kg** Mishra et al. (2016)	-	Pet. Ether extract shows anti-asthmatic potential by mast cell degranulation. **Dose: 50 and 100 mg/kg** Vadnere et al. (2009)
C3.8	*Lepidium sativum* L./Chansur/Cruciferae/Whole plants	Protein extract of lepidium sativum alter the proliferation induced by Con-A. Daoudi et al. (2013)	-	Isolated fractions from ethanol extract of whole plant inhibit bronchospasm induced by histamine and acetylcholine. Rehman et al. (2012), Prasad et al. (2009)
C3.9	*Leptadenia reticulata* (Retz.) Wight and Arn./Meethi dodi)*/*Apocynaceae*/*Root, stem bark	Alcoholic extract increased haematological profile, GSH, SOD, CAT activity and decreased LPO levels in cyclophosphamide-induced rats. **Dose: 100–200 mg/kg** Pravansha et al. (2012)	-	Ethyl acetate fraction inhibit pro-inflammatory cytokines (IL-2, IL-6, TNF-α) and release of prostaglandin to prevent inflammation. **Dose: 600 mg/kg** Mohanty et al. (2015)
C3.10	*Magnolia officinalis* var. officinalis/Himchampa/Magnoliaceae/Bark	-	Isolated compound magnolol and honokiol from petroleum ether extract of bark provoked IRF7 transcripts (magnolol) and reinforcing the host antiviral response via NF-κB pathways (Honokiol). **Dose: 35 mg/L** Chen et al. (2017)	Aqueous extract exhibits anti-allergic actions through inhibition of local immunoglobulin E, histamine release and TNF-α production in 48/80 induced systemic anaphylaxis in rats. **Dose: 0.001–1 g/kg** Shin et al. (2001). Polyphenolic rich extract of *Magnolia officinalis* suppressed the production of inflammatory mediators, NO, pro-inflammatory cytokines, TNF-α and IL-6, and inhibition of TLR3 and NF-κB activation. **Dose: 10 and 200 mg/kg** Fang et al. (2015)
C3.11	*Mucuna pruriens* (L.) DC./Kaunchbeej/Fabaceae/Seeds	*M. pruriens* modulate the immune components like TNF-a, IL-6, IFN-l, IL-1b, iNOS and IL-2. Rai et al. (2017) Alcoholic extract of root influenced both humoral and cell mediated immunity. **Dose: 100, 200 and 400 mg/kg** Murthy and Mishra (2016)	-	Alcoholic extract of seeds of *M. pruriens* act on opoid receptor that located on airway passage and produce inhibitory effect. **Dose: 500 mg/kg** Nuzhat et al. (2013)
C3.12	*Piper betle* L./Paan/Piperaceae/Leaves	Alcoholic extract of *P. betle* leaves showed lymphocyte proliferation, interferon- γ receptors and the pro-duction of nitric oxide. It suppressed phytohaemagglutinin stimulated peripheral blood lymphocyte proliferation. **Dose: 500 mg/kg** Kanjwani et al. (2008)	-	Alcoholic extract of leaves decreased histamine and GM-CSF produced by an IgE-mediated hypersensitive reaction, and inhibited eotaxin and IL-8 secretion in a TNF-α and IL-4-induced allergic reaction. **Dose: 10 mg/ml** Wirotesangthong et al. (2008)
C3.13	*Sesamum indicum* L./Tila*/*Pedaliaceae*/*Seed	Essential oil suppress cellular immunity with the domination of Th2 responses and also modulate macrophages, dendritic cells proinflammatory functions. Dose:100 μg/ml Khorrami et al. (2018)	-	Aqueous extract reduce LPS induced inflammatory gene expression. **EC**_**50**_ **: 100 ng/ml** Deme et al. (2018)
C3.14	*Sida cordifolia* L./Beejband/Malvaceae/Seeds	*S. cordifolia* increased production of T-cell precursor and passive influences on the production of cytokines. **Dose: 2 gm/kg** Tekade et al. (2008)	-	Alcoholic extract of seed inhibit paw edema and granuloma formation. **Dose: 200 and 400 mg/kg** Singh S. et al. (2011)
C3.15	*Swertia chirayita* (Roxb.) H.Karst./Chirayata*/*Gentianacea/Whole plant	-	Chloroform extract inhibit expression of viral protein R in hela cells harboring the TREx plasmid encoding full-length vpr (TREx-HeLa-vpr cells. **Dose: 10 µM** Woo et al. (2019)	Chloroform fraction exhibits bronchodilator effect by Ca^2+^ channel blockade. **Dose: 0.1–3.0 mg/ml** Khan et al. (2012)
C3.16	*Tachyspermum ammi* (L.) sprague/Ajwain/Umbelliferone/Seed	Isolated glycoprotein from aqueous extract of seed proliferate B-cell enriched murine splenocytes and activated macrophages in releasing NO and promoted phagocytosis. **Dose: 1 μg/ml** Shruthi et al. (2017)	Seed oil neutralize antibody for Japanese encephalitis virus. **Dose: 0.5 mg/ml** Roy et al. (2015)	-
C3.17	*Tylophora indica* (Burm.f.) Merr.*/*Antamool/Asclepiadaceae/Roots, leaves	Alkaloidal fraction inhibit proliferation of splenocytes and both macrophages and T cells were found to be vulnerable to tylophora alkaloids. Ganguly et al. (2001)	-	Alcoholic extract of aerial part showed anti-inflammatory effect against carrageenan induced paw oedema and cotton pellet induced granuloma. **Dose: 100, 200 and 400 mg/kg** Raj et al. (2006)
C3.18	*Viola odorata* L./Banafsha/Violaceae/Flowers	-	*V. odorata* effective against multiple protease inhibitors Gerlach et al. (2019)	Aqueous flower extract effectively reduced the hemorrhage area, alveolar wall thickness and septum rupture, and alteration of the epithelial lining of bronchioles of lungs. **Dose: 50 mg/kg** Koochek et al. (2003)

C1: Category 1, includes 21 “Most promising drugs” which have already shown activity against Coronaviruses/HIV/Dengue viruses with their immunomodulatory and anti-allergic/anti-inflammatory properties.

C2: Category 2, composed of 44 “Equally promising drugs” which reportedly have shown anti-viral, immunomodulatory and anti-allergic/anti-inflammatory activities.

C3: Category 2, represents 18 *“Possibly promising drugs”* which have been reported to show anti-viral, immunomodulatory and anti-allergic/anti-inflammatory activities.

#### 3.2.7 Khameera Marwareed

Khameera marwareed is a compound, sugar-based, semisolid Unani formulation used as an immunomodulator. It has been reported to stimulate the immune system through T helper 1 (Th1) type cytokine response and maintains the body in a healthier position to fight against viral infections (Khan et al., 2009). Its ingredients showed powerful antiviral activities by inhibiting replication (Benencia and Courrèges, 1999).

#### 3.2.8 Asgandh Safoof

Asgand (*Withania somnifera* (L.) Dunal) is a very popular Indian medicinal plant. The root powder is used in the Unani system of medicine as an immunomodulator. It is reported that the root’s extract significantly increases the CD4^+^ and CD8^+^ counts (Bani et al., 2006) and blood profile, especially WBC and platelet counts (Agarwal et al., 1999). Aqueous suspension showed potent inhibitory activity toward mitogen-induced proliferative response of T-lymphocytes and prevent SARS-CoV-2 entry by disturbing connections between viral S-protein receptor binding domain and host ACE2 receptor (Balkrishna et al., 2020). The above literature supports the preventive use of Asgandh safoof against COVID-19.

#### 3.2.9 Habb-e-Bukhar

Habb-e-Bukhar is a polyherbal tablet formulation of Unani system of medicine, prescribed in elephantiasis and malarial fever. The main ingredient of Habb-e-Bukhar is cinchona bark. Its active constituent quinine is being used by some countries as either experimental treatment or suggested as a drug with a promising profile against COVID-19 (Devaux et al., 2020). Another constituent, *Tinospora cordifolia* (Willd.) Miers is reported as potent antiviral agent against HSV (Pruthvish and Gopinatha, 2018) as well as suggested for immune-enhancing activity (Rastogi et al., 2020)*.* Thus, literature supports Habb-e-Bukhar in the treatment of COVID-19.

#### 3.2.10 Sharbat-e-Toot Siyah

Sharbat-e-Toot Siyah is composed of the juice of *Morus nigra* L. in a sugar base and is used to treat tonsillitis and sore throat. It has been reported as anti-inflammatory and analgesic and inhibits the pro-inflammatory cytokines (Chen et al., 2016). Very recently, it has been reported to enhance immunomodulatory activity (Lim and Choi, 2019).

#### 3.2.11 Laook-e-Katan

Laook-e-Katan is a sugar-based semisolid Unani formulation composed of *Linum usitatissimum* L. seed, which contains alpha linolenic acid and has been reported to have antiviral, anti-inflammatory, and immunomodulatory activities (Leu et al., 2004; Erdinest et al., 2012; Miccadei et al., 2016). In Unani, it is recommended for respiratory disorders (Table 3).

### 3.3 Siddha Approaches

#### 3.3.1 Nilavembu Kudineer

Nilavembu Kudineer is a polyherbal Siddha formulation prescribed for the prevention and management of viral infections and fevers. It acts as an immunomodulator and plays a defending role against dengue fever and chikungunya. Recent studies showed that formulation has antiviral and antimicrobial actions, which makes it suitable for viral fevers, malaria, and typhoid fever (Mahadevan and Palraj, 2016). Previously, studies proved that most of its constituents are effective as antiviral, anti-asthmatic, and immunobooster agents (Carrasco et al., 2009; Wang et al., 2010; Jin et al., 2011; Chang et al., 2013; Wintachai et al., 2015; Mair et al., 2016).

#### 3.3.2 Ahatodai Manapagu

Ahatodai Manapagu is composed of *Adathoda vasica* Nees leaves, which contains alkaloids like vasicine, the active ingredient in various cough syrups. *A. vasica* has been used in the Indian medicinal system for thousands of years, to treat various types of respiratory disorders (Sampath Kumar et al., 2010). Vinothapooshan et al. suggested that its extract positively modulates the immunity of the host (Vinothapooshan and Sundar, 2011).

#### 3.3.3 Kabasura Kudineer

Kabasura Kudineer is a traditional formulation used in the Siddha system of medicine for managing common respiratory complaints such as flu and cold. Siddha practitioners also recommended this formulation for severe phlegm, dry cough, and fever. It is made up of more than ten herbal ingredients, and each ingredient has a unique pharmacological activity in respiratory disorders. Hence, the ministry of AYUSH recommends its use for symptomatic management in COVID-19 (Sampath Kumar et al., 2010; Jin et al., 2011; Vinothapooshan and Sundar, 2011; Chang et al., 2013).

In addition to Ayurvedic, Unani, and Siddha formulations recommended by AYUSH there are some homeopathic formulations such as Arsenium album, Brayonia alba, and Rhus toxicodendrum have been recommended which have not been included due to controversies over the use of homeopathic medicine**.** These formulations are prepared by dilutions in such a way so that no single detectable molecule is present in the final formulation, which results in controversy (Ernst, 2010). The criticism is due to nonevidential rationale to determine the biological effects of solutions containing unmeasurable starting material (Kaur, 2013).

Further, advancements in pathogenesis and understanding of diseases provide a wider platform to report the pharmacological limitations and opportunities of these highly diluted homeopathic medicines. Day by day, it is becoming more challenging for a pharmacologist to validate the therapeutic claims of homeopathic medicines through experiments. Low acceptance of homeopathic formulations is due to the absence of standardized protocols to justify their pharmacological potential. A major concern is to develop evidence-based validated methods and advancements in the homeopathic system to justify its measurable dilutions, which will help in understanding the mechanism of action and acceptability of homeopathic medicine (Table 4).

### 3.4 Routinely Used Common Indian Medicinal Plants for Exploring Against COVID-19

Ashwagandha, giloe, ginger, cinnamon, tulsi, black pepper, black cumin, amla, turmeric, garlic, and flax seeds have been traditionally used as herbal remedies for multiple diseases since ancient times. These herbs have been utilized in food preparations and traditional medicines in several countries. However, in India, their culinary use is very common and they are a part of kitchen in every house. Similarly, there are some traditional Indian formulations such as Chyawanprash, Triphala, and Rooh Afza etc. that are very commonly used in Indian territory as a part of daily used nutritional supplements. These plants and formulations are very common and at least one of them is being used daily by every Indian, irrespective of religion/community/financial status. The above-mentioned herbs and formulations have been proved potent scientifically for their immunomodulatory, antioxidant, and anti-infective properties, which might be one of the reasons behind the lower death rate of Indians per million of population due to COVID-19 even with minimum health infrastructure.

#### 3.4.1 *Allium sativum* L. (Garlic)

Various research has been conducted *in vivo* to highlight the effect of *A. sativum* in immunomodulation using garlic oil extract. The results showed reduction in serum TNF-α, ICAM-1 and immunoglobulin (G and M) levels confirming the enhancement in immune system activity (Kamel and El-Shinnawy, 2015). Pre-treatment with aqueous garlic extract showed notable antiviral effects mainly by reduction in infectivity and titer of virus against the velogenic strain of Newcastle disease virus in embryonated chicken eggs (Arify et al., 2018). *A. sativum* also showed antiviral effect against avian influenza virus H_9_N_2_ on Vero cells (Rasool et al., 2017). Its defensive effect on allergen-induced airway inflammation in rodent model showed significant reduction in inflammatory cell count, eosinophil infiltration and serum IgE modulation of Th1, Th2, and Th3 cytokines, upregulation of Th-1, Th-3 and simultaneous down-regulation of Th-2 expression. (Hsieh et al., 2019). Old extract of *A. sativum* showed modulation of airway inflammation established in BALB/c mice by reduction in percentage of eosinophil, lavage and serum IgG1 levels, and perivascular inflammation. The study suggested the attenuation of allergic airway inflammation by aged garlic extract (Zare et al., 2008). It has been found that fresh raw garlic extract showed anti-inflammatory effects by decreasing production of prostaglandin E2 (PGE_2_), IL-6, IL-1β, nitric oxide (NO), and leukotrienes (LT D_4_ and E_4_) in lipopolysaccharide activated RAW264.7 cells (Jeong et al., 2016).

#### 3.4.2 *Cinnamomum verum* J.Presl. (Cinnamon) or *Cinnamomum zeylanicum* Blume

*C. verum* essential oil and powder exhibited anti-oxidant, immunostimulant, and antiviral activity in Newcastle disease virus in chickens mainly by modulating total protein, globulin, total antioxidant capacity, and lysozyme activity, and significantly increased phagocytic activity (Islam et al., 2017). Another study reported that *C. zeylanicum* essential oil when blended with other essential oils showed effective antiviral potential against H1N1 and HSV1 viruses. Reduction in virus infectivity has been observed with 99% at 60-min contact time and more than 99.99% after 60 min for both H1N1 and HSV1 viruses (Brochot et al., 2017). Its bark extract exhibited immunomodulatory activity and significantly increased serum immunoglobulins, phagocytic index, neutrophil adhesion, and antibody titer (Niphade et al., 2009). Procyanidine polyphenols (Type A) extracted from *C. zeylanicum* bark showed anti-inflammatory potential in edema induced by carrageenan (Vetal et al., 2013). Alcoholic extract of bark suppressed intracellular release of TNF-α (murine neutrophils) and leukocytes (pleural fluid) as well as inhibition of TNF-α gene expression in lipopolysaccharide-stimulated human peripheral blood mononuclear cells (Joshi et al., 2010).

#### 3.4.3 *Curcuma longa* L. (Turmeric)

Aqueous extract of *C. longa* decreased relative spleen weight and modulation in hematological changes indicating the potential of *C. longa* as an immunomodulator in cyclophosphamide-immunosuppressed *in vivo* model. The study observed promising effects of turmeric as an immunomodulator by representing spleen cells in younger mice (Mustafa and Blumenthal, 2017). *C. longa* extract also showed antiviral potential against dengue virus in *in vitro* and *in vivo* studies on Huh7it-1 cells and a remarkable reduction in viral load has been observed by in *in vivo* model (Ichsyani et al., 2017). Water and ethanolic crude extracts have been found to be antiviral in H5N1 also showed upregulated TNF-α as well as IFN-β mRNA expression, highlighting its promising role in the inhibition of the replication of viruses (Sornpet et al., 2017). Turmeric extract has been found to be anti-allergic in mice immunized with ovalbumin and alum. Attenuation of food allergy by maintaining balance of Th1/Th2 has been reported. Extract has been found to cause reduction in Th2 and increase in Th1 cell-related cytokines. Further, increased levels of IgE, IgG1 and mMCP-1 levels were also decreased proving effects of turmeric in allergic disorders mainly, asthma and food allergies (Shin et al., 2015). Various other studies also reported anti-inflammatory effects of *C. longa* either alone or in combination (Lee et al., 2020).

#### 3.4.4 *Linum usitatissimum* L. (Flax Seed)

Heteropolysaccharide, extracted from flax seed hull possessed immunomodulatory activity and anti-hepatitis B virus potential. It significantly stimulated mRNA expression of TNF-α, NO and IL exhibiting immune responses in murine macrophages. Antiviral activity has been reported through inhibition of expression of surface antigen as well as envelop antigen and also interfered with DNA replication. The study suggested its promising potential as an immunostimulant and vaccine adjuvant (Liang et al., 2019). It showed anti-inflammatory and immunomodulatory potential in obesity-associated insulin resistance. Its oil in co-culture with 3T3-L1 adipocytes-RAW 264.7 macrophages of C57BL/6 mice reported shifting the cytokines toward anti-inflammatory with a decrement in TNF-α. Immunomodulation has been observed through an increase in levels of Th2-related cytokine (IL-4), serum anti-ova IgG1, and IgE, and a decrease in Th-1 related cytokines (TNF-α and IFN-γ) and anti-ova IgG levels (Palla et al., 2015). Another study reported the immunomodulatory activity of phenolic components of flax seed mainly through reduction in cell-mediated immune responses (Kasote et al., 2012).

#### 3.4.5 *Nigella sativa* L. (Black Cumin)

Nigella sativa L.’s bioactive compounds have been observed as potential inhibitors of COVID-19 in molecular docking studies. Nigellidine gave energy complex at active site (6LU7) with energy scores closest to chloroquine and better than hydroxychloroquine and favipiravir whereas α-hederin gave energy complex at the active site (2GTB) with energy scores better than chloroquine, hydroxychloroquine, and favipiravir (Salim and Noureddine, 2020). The alcoholic seed extract has shown immunosuppressive activity on a phytohemagglutinin and immunostimulating effect on non-phytohemagglutinin (PHA) stimulated proliferation (Alshatwi, 2014). The thymoquinone-rich oil showed suppression of cytokine signaling molecules, and PGE_2_ in T-lymphocytes as well as enhanced PGE2 release in adrenocarcinomic human alveolar basal epithelial A549 cells (Koshak et al., 2018).

#### 3.4.6 *Ocimum sanctum* L. (Tulsi)

Hydro-alcoholic extract of *Ocimum sanctum* inhibited intracellular multiplication of virus. It also inhibits non-specific interference with virus-cell interactions in H9N2 viruses. (Ghoke et al., 2018). The immunomodulatory potential of alcoholic leaves extracts at IC_50_ value of 73.3 μg/ml showed reduction in hepatic parasite and, skewing of the humoral response toward Th1 type (Bhalla et al., 2017). *O. sanctum* inhibits leukotriene-C4-synthase, leukotriene-A4-hydrolase and cyclooxygenase-2 activities in cultured HL-60 cells and causes a significant reduction in OVA-induced lung inflammation (Soni et al., 2015).

#### 3.4.7 *Phyllanthus emblica* L. (Amla)

Amla has been reported to significantly relieve chromium-induced immunosuppressive effect on lymphocyte proliferation and led to restoration in production of IL-2 and INFγ (Sai Ram et al., 2002). Phenolics from emblica has been found to increase splenocytes proliferation. Geraniin and isocorilagin showed significant immunostimulatory effects (Liu et al., 2012). Ethanolic extract of amla strongly reduced levels of pro-inflammatory cytokines and increased levels of anti-inflammatory cytokine (Bandyopadhyay et al., 2011). An isolated compound (1, 2, 4, 6-tetra-*O*-galloyl-β-d-glucose) of *P. emblica* showed antiviral potential against HSV by HSV-1 inactivation, which leads to inhibition of early infection indulging attachment and penetration of virus, suppression of intracellular growth and inhibited gene expression of HSV-1 E and L along with DNA replication (Xiang et al., 2011).

#### 3.4.8 *Piper nigrum* L. (Black Pepper)

Piperamides isolated from *P. nigrum* fruits showed significant inhibition of coxsackie virus type B3 in a cytopathic effect inhibition assay (Mair et al., 2016). Aqueous extract of *P. nigrum* acted as a potent modulator of the macrophages and significantly enhanced splenocyte proliferation in a dose-dependent manner (Majdalawieh and Carr, 2010). The isolated alkaloid from *P. nigrum* exhibited anti-inflammatory effect in RAW 264.7 cells stimulated by LPS and significant inhibition in iNOS-mediated NO and IL-1β, IL-6, and TNF-α. It also demonstrated anti-inflammatory activity in edema induced by carrageenan (Pei et al., 2020). Reports have confirmed the improvement of ovalbumin-induced nasal epithelial barrier dysfunction in allergic rhinitis mouse model. Further, protection of epithelium integrity, enhancement in E-cadherin tight junction protein as well as inhibition of the degraded levels of zonula occludens-1 and occluding in the nasal passage have been reported. Additionally, enhancing the activation of Nrf2/HO-1 signaling showed anti-allergic and anti-asthma activities (Bui et al., 2020).

#### 3.4.9 *Tinospora cordifolia* (Willd.) Miers (Giloe)

*In vitro* screening of *T. cordifolia* silver nanoparticles against chikungunya virus cell showed significant antiviral potential (Sharma V. et al., 2019). Alcoholic leaves extract of *T. cordifolia* significantly decreases intracellular reactive oxygen species (ROS) in chikungunya patients with high levels of intracellular ROS in persisting polyarthralgia by *ex vivo* treatment (Banerjee et al., 2018). An *in vitro* study revealed the antiviral potential of crude stem extract of *T. cordifolia* against HSV in Vero cell lines by inhibiting the growth of HSV (Pruthvish and Gopinatha, 2018). Aqueous extract of *T. cordifolia* stem significantly increase INFγ and IL levels (IL-1, IL-2, IL-4) in isolated chicken peripheral blood mononuclear cells (PBMCs) against infectious bursal disease virus. Further, immunomodulatory potential via the toll like receptor (TLR)-mediated pathway was also concluded (Sachan et al., 2019). The hydro-alcoholic extract of *T. cordifolia* stem in drinking water caused enhancement of cellular immunity as well as humoral immunity in broiler chicks (Nety et al., 2017). Chloroform extract significantly prevented pro-inflammatory biomarkers (IL-6, IL-1β and PGE2) and decreased paw oedema (*p* ≤ 0.05) with no toxicity reported when conducted in RAW264.7 macrophages (Philip et al., 2018).

#### 3.4.10 *Withania somnifera* (L.) Dunal (Ashwagandha)

Multiple studies have proved that Ashwagandha has antiviral and immunomodulatory potential. Very recently, an *in silico* study concluded that Withaferin-A exhibits antiviral potential against SARS-CoV-2 through inhibiting RNA polymerase with higher binding energy than hydroxychloroquine and other drugs used against SARS-CoV-2. Another study on withanone showed blockage of SARS-CoV–2 entry and also its subsequent infection by interrupting electrostatic interactions between the RBD and ACE2 (Balkrishna et al., 2020). Grover and colleagues through molecular docking reported the potential of withaferin A against HSV through inhibition of DNA polymerase enzyme (Grover et al., 2011). *W. somnifera* molecular mechanism has been elucidated by using network ethnopharmacological technique and reported that withanolide-phytosterol combination is a good immunomodulator (Chandran and Patwardhan, 2017). *W. somnifera* formulation (supplemented with minerals) has been reported to improve both cellular and humoral immunity as well as hematological profile in addition to the significant inhibition in mouse splenocytes (Trivedi et al., 2017). Aqueous root extract of *W. somnifera* attenuates production of pro-inflammatory cytokines and transcription factor in collagen-induced arthritis (Khan et al., 2018). A study in 2018 showed that *W. somnifera* significantly inhibited mRNA expression of inflammatory cytokines and promotes the mRNA expression of the anti-inflammatory cytokine in HaCaT cells (Sikandan et al., 2018).

#### 3.4.11 *Zingiber officinale* Roscoe (Ginger)

Fresh ginger aqueous extract showed antiviral activity against human respiratory syncytial virus in human respiratory tract cell lines (HEp-2 and A549) and decreased the plaque counts in a dose-dependent manner. It also stimulated the secretion of IFN-β that contributes to counteracting against viral infection (Chang et al., 2013). It also showed antiviral potential against avian influenza virus H9N2 on Vero cells in a dose-dependent manner (Rasool et al., 2017). Oral administration of Soft gel capsules containing a *Z. officinale* in combination showed immunomodulatory and anti-inflammatory properties parallel to those exerted by positive control, and gene expression data highlighted overall same transcriptional remodeling (Dall’Acqua et al., 2019). A study on essential oil of ginger reported immunomodulatory effects by improving the humoral immunity in cyclophosphamide-immunosuppressed mice in a dose-dependent manner (Carrasco et al., 2009). Oral administration of alcoholic ginger extract to allergic rhinitis patients showed significant reduction in total nasal symptom scores (TNSS), with overall improvement in rhino conjunctivitis quality of life questionnaire (Yamprasert et al., 2020). The aqueous and alcoholic extracts of rhizome decreased goblet cell hyperplasia, infiltration of inflammatory cells in airways with reduced total and differential counts of eosinophils and neutrophils in mouse model (Khan et al., 2015) (Table 5).

### 3.5 Routinely Used Indian Natural Health Supplements to Explore for Use Against COVID 19

#### 3.5.1 Chyawanprash

Chyawanprash is an Ayurvedic polyherbal health supplement, which is made up of concentrated extracts of nutrient-rich herbs and minerals. Chyawanprash comes under Awaleha (electuaries/herbal jams) due to its consistency, and composed of Amla fruit as a base, which is considered as the most active Rasayana to improve strength, stamina, and vitality.

Although several types of research have been published on Chyawanprash to report its health benefits against various ailments, the study reports antioxidant (Anil and Suresh, 2011) free radical scavenging (Bhattacharya et al., 2002) antibacterial, antiviral, anti-inflammatory, antiallergic, and antithrombotic effects (Gupta et al., 2017). In a randomized controlled trial, it was found effective for pulmonary tuberculosis as an adjunct to antitubercular drugs. (Debnath et al., 2012; Sharma R. et al., 2019). An experimental study showed that Chyawanprash pre-treatment reduced plasma histamine levels and IgE release when rats and mice were challenged with allergen- and ovalbumin-induced allergy, suggesting its anti-allergic potential. NK cell activity was significantly increased by Chyawanprash treatment. On treating dendritic cells with Chyawanprash, there was a significant increase in immunity marker levels as well as phagocytic activity that proves its immunomodulatory activity (Sastry et al., 2011).

#### 3.5.2 Triphala

Triphala is a well-known polyherbal Ayurvedic medicine consisting of equal proportions of fruits of *Phyllanthus emblica* L., *Terminalia bellerica* (Gaertn.) Roxb. and *Terminalia chebula* Retz. in the form of powder for digestive and refreshing action. Triphala is associated with many of the therapeutic potentials such as antioxidants, antiinflammatory, antineoplastic, antimicrobial, antidiabetic, etc. (Peterson et al., 2017). Alcoholic extract of Triphala showed specific antimicrobial activity (Tambekar and Dahikar, 2011), broad-spectrum antimicrobial activity against antibiotic-resistant bacteria isolated from humans (Peterson et al., 2017).

Triphala extract was found more active than the NSAID drug, indomethacin, in improving arthritic and inflammatory effects and reduced expression of inflammatory mediators through inhibition of NF-κB activation (Kalaiselvan and Rasool, 2015). In LPS-stimulated macrophages, Triphala inhibited the production of inflammatory mediators, intracellular free radicals, and inflammatory enzymes (Reddy et al., 2009; Kalaiselvan and Rasool, 2016). It has been shown to reduce multiple cell signaling pathways of inflammation and oxidative stress and prevented the noise-stress induced changes in rats thereby strengthening the cell-mediated immune response (Prasad and Srivastava, 2020). A clinical study of Triphala showed immunostimulatory properties on T cells and NK cells, however did not change the cytokine levels in healthy volunteers (Phetkate et al., 2012). The individual constituents of Triphala have also showed immunomodulatory activity (Aher and Wahi, 2011). The stated data on Triphala reveals that it is a powerful polyherbal formulation with countless therapeutic uses for maintaining homeostasis as well as the cure and management of various disease.

#### 3.5.3 Sharbat Rooh Afza

Rooh Afza is a well-known refreshing formulation with global acceptance. It is a concentrated squash prepared as sugar syrup with distillates of numerous medicinal plants including seeds of khurfa (*Portulaca oleracea* L.), kasni (*Cichorium intybus* L.), angoor (*Vitis vinifera* L.), nilofar (*Nymphaea alba* L.), Neel Kamal (*Nymphaea nouchali* Burm. f.), kamal (*Nelumbo nucifera* Gaertn.)*,* gaozaban (*Borago officinalis* L.), badiyan (*Coriandrum sativum* L.), fruits/juices of santara (*Citrus* × *sinensis* (L.) Osbeck), ananas (*Ananas comosus* (L.) Merr.), seb (*Malus domestica* (Suckow) Borkh.), berries (*Rubus fruticosus* L.), vegetables like palak (*Spinacia oleracea* L.), gazar (*Daucus carota* L.), and pudina (*Mentha arvensis* L.). Rooh Afza boosts the energy system of the body by naturally refreshing. Although there is no evidence on Rooh Afza revealing its therapeutic value, its constituents have been reported as potently antiviral, immunomodulatory, and antiallergic against respiratory disorders.

The flower extract of *P. oleracea* possessed significant antioxidant and protective effects against DNA damage induced by necrotic effects (Dogan and Anuk, 2019). *V. vinifera* fruits exhibit anti-asthmatic activity by inhibiting cellular response and subsequent production of inflammatory cytokines (Arora et al., 2016). A study on *N. alba* flower has been reported against inflammatory activity in Swiss Albino mice using acute inflammatory models in a dose-dependent manners (RS et al., 2013). The immunoregulatory and anti-HIV-1 enzyme activities of *N. nucifera* suggest that it could be potentially important against virus development (Jiang et al., 2011).

Thus, it can be perceived that Rooh Afza not only provides natural refreshness to the body but also has antioxidant, immunomodulatory, and anti-inflammatory/antiviral activities. However, to validate the scientific data on the therapeutic value of Rooh Afza, experimental research should be undertaken to prove its role in health benefits therapeutically.

The above studies encourage further investigations of traditional medicinal plants for their preventive use against coronavirus infection. The herbs could be taken individually or synergistically at appropriate concentrations as candidates for developing potential therapeutic tools against COVID-19.

### 3.6 Potential Indian Medicinal Plants for Exploring Against COVID-19

There are many other Indian medicinal plants, which are either part of AYUSH recommendations as such or as ingredients of formulations or are known for improving immunity with antiviral and anti-allergic/anti-inflammatory potential and can offer potential leads against COVID-19. Table 5 provides a list of 83 medicinal plants categorized on a priority basis as per their reported properties. Category 1 (C1) includes 21 “*Most promising drugs”* which have already shown activity against Coronaviruses/HIV/Dengue viruses with their immunomodulatory and anti-allergic/anti-inflammatory properties. Category 2 (C2) is composed of 44 *“Equally promising drugs”* which reportedly have shown anti-viral, immunomodulatory, and anti-allergic/anti-inflammatory activities. Category 3 (C3) represents 18 *“Possibly promising drugs”* which have been reported to show anti-viral/immunomodulatory and/or anti-allergic/anti-inflammatory activities.

Listed medicinal plants and AYUSH recommended formulations could help as the potential alternate therapeutics for management and cure of COVID-19. However, this needs scientific explorations and validation of their preclinical and clinical studies. Since there is such a rich diversity, many other medicinal plants and their bioactive fractions need the attention of the scientific community to be explored against COVID-19.

## 4 Conclusion

The SARS-CoV-2 has become a threat to human population due to non-availability of approved vaccines or drugs for its treatment. Many herbs that have been reported to work as an immunity booster against other viral infections, and to possess anti-allergic/anti-inflammatory activities, need to be tested against COVID-19. Indian Traditional Medicines have a wide potential for being used in these tough times either for prophylaxis or as adjuvant, owing to their longstanding use in community, ancient references and scientific evidence about their safety and clinical efficacy. The AYUSH ministry, Govt of India has issued several advisories from time to time, considering the strength and evidence of these systems of medicines and making considerable efforts to encourage researchers to explore herbal products for COVID-19. Interventions and herbal formulations from different AYUSH systems have the support of evidence for their immunity-enhancing, anti-inflammatory and antiviral effects. These herbal remedies may, therefore, provide some respite until the availability of trial-tested drug or vaccine to combat the COVID-19 menace. Further, it was noted that a major portion of public and private funding were dedicated to AYUSH trials. More than 50% of these trials were sponsored by the government and various stakeholders associated with the Ministry of AYUSH. It is expected that the results of these clinical studies will be disseminated soon at the public platform so that the policymakers from the AYUSH systems of medicines may reframe their policies for public health and provide information to the global scientific community, which could form a platform for collaborative studies at the national and global levels. The medicinal plant species discussed in this review and categorized for their preclinical and clinical investigation may be taken up by research organizations on priority basis, as this may result in the development of lead molecule against SARS-CoV-2 and COVID-19. Keeping in view the potential of AYUSH medicines and medicinal plants of India, the herbal drug, manufacturers, and the national and global research organizations should develop necessary strategies for furtherance of preclinical and clinical research on these promising therapeutic leads.
